# Number needed to treat (NNT) in clinical literature: an appraisal

**DOI:** 10.1186/s12916-017-0875-8

**Published:** 2017-06-01

**Authors:** Diogo Mendes, Carlos Alves, Francisco Batel-Marques

**Affiliations:** 1 AIBILI – Association for Innovation and Biomedical Research on Light and Image, CHAD – Centre for Health Technology Assessment and Drug Research, Azinhaga de Santa Comba, Celas, 3000-548 Coimbra, Portugal; 2 University of Coimbra, School of Pharmacy, Laboratory of Social Pharmacy and Public Health, Coimbra, Portugal

**Keywords:** Numbers needed to treat, Evidence-based medicine, Epidemiologic methods, Data interpretation, Statistical, Meta-analysis, Randomized controlled trial, Cohort studies, Case–control studies

## Abstract

**Background:**

The number needed to treat (NNT) is an absolute effect measure that has been used to assess beneficial and harmful effects of medical interventions. Several methods can be used to calculate NNTs, and they should be applied depending on the different study characteristics, such as the design and type of variable used to measure outcomes. Whether or not the most recommended methods have been applied to calculate NNTs in studies published in the medical literature is yet to be determined. The aim of this study is to assess whether the methods used to calculate NNTs in studies published in medical journals are in line with basic methodological recommendations.

**Methods:**

The top 25 high-impact factor journals in the “General and/or Internal Medicine” category were screened to identify studies assessing pharmacological interventions and reporting NNTs. Studies were categorized according to their design and the type of variables. NNTs were assessed for completeness (baseline risk, time horizon, and confidence intervals [CIs]). The methods used for calculating NNTs in selected studies were compared to basic methodological recommendations published in the literature. Data were analyzed using descriptive statistics.

**Results:**

The search returned 138 citations, of which 51 were selected. Most were meta-analyses (*n* = 23, 45.1%), followed by clinical trials (*n* = 17, 33.3%), cohort (*n* = 9, 17.6%), and case–control studies (*n* = 2, 3.9%). Binary variables were more common (*n* = 41, 80.4%) than time-to-event (*n* = 10, 19.6%) outcomes. Twenty-six studies (51.0%) reported only NNT to benefit (NNTB), 14 (27.5%) reported both NNTB and NNT to harm (NNTH), and 11 (21.6%) reported only NNTH. Baseline risk (*n* = 37, 72.5%), time horizon (*n* = 38, 74.5%), and CI (*n* = 32, 62.7%) for NNTs were not always reported. Basic methodological recommendations to calculate NNTs were not followed in 15 studies (29.4%). The proportion of studies applying non-recommended methods was particularly high for meta-analyses (*n* = 13, 56.5%).

**Conclusions:**

A considerable proportion of studies, particularly meta-analyses, applied methods that are not in line with basic methodological recommendations. Despite their usefulness in assisting clinical decisions, NNTs are uninterpretable if incompletely reported, and they may be misleading if calculating methods are inadequate to study designs and variables under evaluation. Further research is needed to confirm the present findings.

**Electronic supplementary material:**

The online version of this article (doi:10.1186/s12916-017-0875-8) contains supplementary material, which is available to authorized users.

## Background

The concept of ”number needed to treat” (NNT) was introduced in the medical literature by Laupacis et al. in 1988 [1]. NNT is an absolute effect measure which is interpreted as the number of patients needed to be treated with one therapy versus another for one patient to encounter an additional outcome of interest within a defined period of time [1, 2]. The computation of NNT is founded on the cumulative incidence of the outcome per number of patients followed over a given period of time, being classically calculated by inverting absolute risk reduction (ARR) (also called risk difference [RD]) between two treatment options [1, 2].

Some characteristics are inherently associated with the concept of NNT. The resulting value is specific to a single comparison between two treatment options within a single study, rather than an isolated absolute measure of clinical effect of a single intervention. Thus, NNT is specific to the results of a given comparison, not to a particular therapy [3]. In addition, three other factors, beyond the efficacy or safety of the intervention and the comparator, influence NNT: baseline risk (i.e., control event rate [CER]), time frame, and outcomes [3].

The use of NNT has been valuable in daily clinical practice, namely at assisting physicians in selecting therapeutic interventions [4, 5]. Further, this metric has the potential for use as a supportive tool in benefit-risk assessments and in helping regulators make decisions on drug regulation [6–8].

The Consolidated Standards of Reporting Trials (CONSORT) statement recommends the use of both relative and absolute measures of effect for randomized controlled trials (RCTs) with binary and time-to-event outcomes [9, 10]. The *British Medical Journal (BMJ)* requires that, whenever possible, absolute rather than relative risks and NNTs with 95% confidence intervals (CIs) are to be reported in RCTs [11]. Yet, few authors express their findings in terms of NNT or ARR [12–14]. Relative effect measures, such as relative risk (RR) or odds ratio (OR), are more commonly seen in the scientific literature [14, 15]. Despite the unquestionable usefulness of relative effect measures, they do not reflect baseline risks, making it impracticable to discriminate large from small treatment effects, and leading sometimes to misleading conclusions [15–17].

Although the NNT was originally conceived to be used in RCTs [1], the concept has been used to express treatment differences in comparative studies with other designs, including systematic reviews and meta-analyses, and observational studies (cohort and case–control studies) [18–23]. Note that the terms ”number needed to treat to benefit” (NNTB) and ”number needed to treat to be harmed” (NNTH) were proposed to distinguish between beneficial and harmful outcomes, respectively [24]. Furthermore, “number needed to be exposed” (NNE) has been proposed to apply the concept of NNT in observational studies, in which the focus is exposure rather than treatment [22]. NNEB and NNEH can be used to describe the number needed to be exposed for one person to benefit or be harmed [22]. In order to simplify, the term NNT is used throughout this paper.

The calculation of NNT should be based upon the use of methods that align with the characteristics of a given study, such as the research design and the type of variable (e.g., binary, time to event, or continuous) used to express the outcome of interest [19, 22, 25–32]. The use of inadequate methods may lead to erroneous results [12, 29, 30, 33, 34]. A previous research study analyzing articles published in four major medical journals found that NNTs were miscalculated in 60% of RCTs involving varying follow-up times [29]. The authors of another paper concluded that 50% of the RCTs reporting NNTs derived from time-to-event outcomes applied inadequate calculation methods [12]. Moreover, only 34% of RCTs presented the corresponding CIs for point-estimate NNTs [12]. The application of inadequate methods within other research designs, such as using pooled RDs in meta-analyses [35, 36] or unadjusted incidence rates in observational studies [22, 34], has also been pointed out.

The main goal of this study is to assess whether the methods used to calculate NNT in studies published in medical journals are in line with basic methodological recommendations.

## Methods

### Studies reporting NNT in medical journals

#### Identification and selection of studies

PubMed was searched for papers reporting NNT estimates that were published between 2006 and 2015 in the top 25 high-impact factor journals in the category of “General and/or Internal Medicine,” according to the Science Citation Index (Additional file 1: Table S1) [37]. The search was restricted to these journals because they are more likely to influence clinicians’ perceptions on the benefits and harms of medicines [38]. No further limits were used in the search strategy (Additional file 1: Table S2).

Titles and abstracts of all retrieved citations were screened by two independent reviewers (DM and CA) to identify potentially relevant publications. Full texts were retrieved for relevant citations. Discrepancies were resolved by majority decision (two of three) involving a third investigator (FBM).

Studies were included if they met the following inclusion criteria: (1) have a control group; (2) assess the effect of a pharmacological intervention on beneficial and/or harmful outcomes; (3) express at least one resulting effect by means of the NNT. Studies assessing medical interventions other than pharmacological interventions (e.g., surgical techniques, dietary interventions, lifestyle modifications) were not included.

### Data extraction

#### General characteristics of included studies

Data elements extracted to describe general study characteristics included: (1) study reference (authors and journal name); (2) year of publication; (3) country (determined by the first author’s affiliation); (4) study design; (5) number of included studies (for systematic reviews and meta-analyses); (6) number of participants; (7) study duration (i.e., length of participants’ follow-up in longitudinal studies); (8) disease/condition of the studied population; (9) pharmacological interventions (including comparators); (10) primary outcome (including its classification as an efficacy and/or safety outcome). Diseases/conditions were classified using the Medical Dictionary for Regulatory Activities (MedDRA), v. 18.0, according to the System Organ Class (SOC) [39].

#### Characteristics of NNTs in included studies

Data were collected from included studies to describe and characterize NNTs as well as to allow for further assessment of calculating methods, according to a list of pre-defined queries (Additional file 1: Table S3 and Table S4). When the methodology used to calculate NNTs was not described in the methods section of the included studies, information from the results or the discussion sections, namely statements given in the text, was used to identify the calculating methods.

### Methods recommended to calculate NNT

#### Methodological recommendations

A summary of basic and general recommendations was set up based upon the evidence reported in the *Cochrane Handbook for Systematic Reviews of Interventions* [31], in a thorough review performed by Bender about methods to obtain NNTs for different study designs [25], and also in another review that focused on observational studies [21]. In addition, a limited, non-systematic literature search was performed in PubMed to identify papers later published that could complement this evidence (Additional file 1: Table S5).

##### Systematic review and meta-analysis

The NNT should be calculated based upon the use of a relative effect because relative effects tend to be more stable across risk groups than absolute differences [19, 31, 40, 41]. The RR and OR, obtained within fixed or random effects regression models, appear to be reasonably constant across different baseline risks [19]. The pooled RR or OR can be used to calculate individualized NNTs for different baseline risks (i.e., *π*_0_ the risk control group), using formulas (1) or (2) [19, 25, 31]. Further, expressing RR or OR as a variety of NNTs across a range of different baseline risks has been recommended [18, 31, 36].

1 $$ N N T=\frac{1}{\left(1- RR\right)\times {\pi}_0},\kern0.5em  f o r\kern0.5em  R R<1;\kern0.5em  N N T=\frac{1}{\left( RR-1\right)\times {\pi}_0},\kern0.5em  f o r\kern0.5em  R R>1 $$

2 $$ N N T=\frac{1}{\left(1- OR\right)\times {\pi}_0}+\frac{OR}{\left(1- OR\right)\times \left(1-{\pi}_0\right)},\kern0.5em  f o r\kern0.5em  O R<1;\kern0.5em  N N T=\frac{1}{\left( OR-1\right)\times {\pi}_0}+\frac{OR}{\left( OR-1\right)\times \left(1-{\pi}_0\right)},\kern0.5em  f o r\kern0.5em  O R>1 $$

##### Randomized controlled trials

In RCTs with a binary outcome and a defined period of time during which all patients are followed, the NNT is estimated based upon the use of simple proportions of patients with the outcome (i.e., *π*_0_ the risk control group and *π*_1_ the risk in treatment group), according to formula (3) [1, 2]:

3 $$ N N T=\frac{1}{\pi_1-{\pi}_0}=\frac{1}{RD} $$

In RCTs with time-to-event outcomes, the time of follow-up is not equal for all patients. Simple proportions should not be used to estimate NNTs because they do not account for varying follow-up times [25, 29]. In such studies, the Kaplan-Meier approach can be used to estimate proportions of patients with the outcome of interest over time [26]. The NNT can then be calculated by inverting the RD between cumulative incidences (i.e., survival probabilities *S*_1_(*t*) for treatment groups and *S*_0_(*t*) for control group) at a given point of time (*t*), as shown in formula (4) [26]:

4 $$ N N T=\frac{1}{S_1(t)-{S}_0(t)} $$

Further, the hazard ratio (HR), estimated by means of the Cox regression model, can be used to estimate the NNT if the assumption of proportional hazards is fulfilled and *S*_0_(*t*) is available, as described in formula (5) [26]:

5 $$ N N T=\frac{1}{{\left({S}_0(t)\right)}^{HR}-{S}_0(t)} $$

##### Observational studies

Due to the lack of randomization, the estimation of treatment effects in observational studies requires adjustment for confounding factors [22]. Regression-based methods, namely multiple logistic regression, or propensity score methods can be performed to estimate adjusted relative effects [21]. The NNT should also be adjusted and not based on crude risk differences without adjustment [22].

##### Case–control studies

In case–control studies, multiple logistic regression is usually performed to estimate adjusted OR as a relative effect measure [22, 23]. The NNT can be calculated by combining the adjusted OR with the risk in control or unexposed group (usually called the unexposed event rate [UER]) [22, 27]. In case–control studies the UER is obtained from an external source (for example, controls in RCTs or unexposed subjects in cohort studies) [27]. Formula (2), where *π*_0_ = UER, should be used to calculate adjusted NNT from adjusted OR. If the relative effect measure is adjusted RR, then formula (1) should be applied.

##### Cohort studies

In cohort studies using regression-based methods, two general approaches can be used to estimate NNT. The first approach is based upon the use of adjusted OR, estimated by means of multiple logistic regression [22]. Adjusted NNT is obtained with the application of adjusted OR to UER, as described in formula (2). However, this approach should only be used if there is a small variation of the risks around the mean [23]. The mean risk of unexposed subjects (UER), which is estimated by means of the logistic regression model, can be used to calculate adjusted NNT for the corresponding confounder profile. Another method that can be used is to calculate NNT for some fixed confounder profiles [22]. In the second approach, NNT is calculated by taking the reciprocal of the average RD over the observed confounder values, estimated by means of multiple logistic regression [23]. In general, the approach based upon the average RD should be applied [23].

For time-to-event outcomes, NNT can be estimated as the reciprocal of the difference between two marginal probabilities, within a given duration of follow-up, using an adjusted survival model (e.g., the Cox proportional hazards regression model) [21, 42–44].

In cohort studies using propensity score methods, NNT can be estimated by inverting RD, which is directly estimated by comparing the probability of the outcome between treated and untreated subjects in the matched sample in propensity score matching [21]. If the outcome is time to event, NNT is given by the reciprocal of the difference estimated from Kaplan-Meier survival curves in treated and untreated subjects within a given duration of follow-up [21].

### Adherence to methodological recommendations

The methods used to calculate NNTs in studies from medical journals were compared to basic methodological recommendations. The adherence of calculating methods to methodological recommendations was assessed, considering the study design and the type of variable used to measure outcomes of interest.

### Data analysis

Data were analyzed using descriptive statistics. Data analyses were performed using Microsoft® Excel® 2013.

## Results

**Fig. 1:**
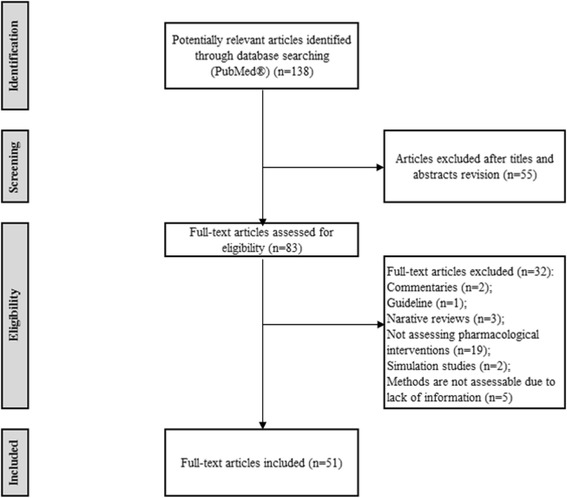
Flow of studies through the review process

Figure 1 presents the search strategy flowchart. From 138 publications, 51 were selected after excluding studies not fulfilling the inclusion criteria.

Table 1 presents a summary of the main characteristics of included studies, namely the characteristics of variables and effect measures used to assess effects of interventions and the completeness of data around NNT estimates. A detailed description of the characteristics of each study is provided in Additional file 1: Table S6.

**Table 1:** Characteristics of the included studies and of the number needed to treat (NNT)

Characteristics	Meta-analysis (*n* = 23)		RCT (*n* = 17)		Cohort (*n* = 9)		Nested case–control (*n* = 2)		Overall (*n* = 51)	
Journal										
*JAMA*	9	(39.1%)	4	(23.5%)	2	(22.2%)	2	(100.0%)	17	(33.3%)
*Lancet*	6	(26.1%)	7	(41.2%)	1	(11.1%)	0	(0.0%)	14	(27.5%)
*Am J Med*	2	(8.7%)	0	(0.0%)	2	(22.2%)	0	(0.0%)	4	(7.8%)
Other	6	(26.1%)	6	(35.3%)	4	(44.4%)	0	(0.0%)	16	(31.4%)
Country										
USA	13	(56.5%)	2	(11.8%)	6	(66.7%)	0	(0.0%)	21	(41.2%)
UK	4	(17.4%)	2	(11.8%)	0	(0.0%)	0	(0.0%)	6	(11.8%)
Canada	1	(4.3%)	2	(11.8%)	1	(11.1%)	2	(100.0%)	6	(11.8%)
Other	5	(21.7%)	11	(64.7%)	2	(22.2%)	0	(0.0%)	18	(35.3%)
Disease/condition										
Infections and infestations	4	(17.4%)	2	(11.8%)	1	(11.1%)	0	(0.0%)	7	(13.7%)
Cardiac disorders	3	(13.0%)	3	(17.6%)	1	(11.1%)	0	(0.0%)	7	(13.7%)
Psychiatric disorders	4	(17.4%)	3	(17.6%)	0	(0.0%)	0	(0.0%)	7	(13.7%)
Other	12	(52.2%)	9	(52.9%)	7	(77.8%)	2	(100.0%)	30	(58.8%)
Primary outcome of study										
Efficacy	12	(52.2%)	16	(94.1%)	2	(22.2%)	0	(0.0%)	30	(58.8%)
Safety	2	(8.7%)	1	(5.9%)	6	(66.7%)	2	(100.0%)	11	(21.6%)
Efficacy and safety	9	(39.1%)	0	(0.0%)	1	(11.1%)	0	(0.0%)	10	(19.6%)
Type of variable (primary outcome)										
Binary	22	(95.7%)^a^	13	(76.5%)	5	(55.6%)	1	(50.0%)	41	(80.4%)
Time to event	1	(4.3%)	4	(23.5%)	4	(44.4%)	1	(50.0%)	10	(19.6%)
Relative effect measure										
Yes										
Relative risk	11	(47.8%)^b^	5	(29.4%)	2	(22.2%)	0	(0.0%)	18	(35.3%)^a^
Odds ratio	9	(39.1%)^b^	4	(23.5%)	2	(22.2%)	1	(50.0%)	16	(31.4%)^a^
Hazard ratio	1	(4.3%)	3	(17.6%)	3	(33.3%)	0	(0.0%)	7	(13.7%)
Rate ratio	0	(0.0%)	0	(0.0%)	1	(11.1%)	1	(50.0%)	2	(3.9%)
No	3	(13.0%)	5	(29.4%)	1	(11.1%)	0	(0.0%)	9	(17.6%)
Outcome expressed with NNT										
Primary outcome	6	(26.1%)	14	(82.4%)	7	(77.8%)	1	(50.0%)	28	(54.9%)
Secondary outcome	0	(0.0%)	2	(11.8%)	0	(0.0%)	0	(0.0%)	2	(3.9%)
Primary and secondary outcomes	17	(73.9%)	1	(5.9%)	2	(22.2%)	1	(50.0%)	21	(41.2%)
NNT for benefit or harm?										
Benefit	8	(34.8%)	15	(88.2%)	3	(33.3%)	0	(0.0%)	26	(51.0%)
Harm	2	(8.7%)	1	(5.9%)	6	(66.7%)	2	(100.0%)	11	(21.6%)
Benefit and harm	13	(56.5%)	1	(5.9%)	0	(0.0%)	0	(0.0%)	14	(27.5%)
Type of NNT calculated in the study										
Person-based NNT	21	(91.3%)^a^	13	(76.5%)	5	(55.6%)	1	(50.0%)	40	(78.4%)
Person-time-based NNT	2	(8.7%)	4	(23.5%)	4	(44.4%)	1	(50.0%)	10	(21.6%)
Completeness of NNT estimate										
Control event rate										
Yes	13	(56.5%)	17	(100.0%)	6	(66.7%)	1	(50.0%)	37	(72.5%)
No	10	(43.5%)	0	(0.0%)	3	(33.3%)	1	(50.0%)	14	(27.5%)
Time horizon										
Yes	10	(43.5%)	17	(100.0%)	9	(100.0%)	2	(100.0%)	37	(72.5%)
No	13	(56.5%)	0	(0.0%)	0	(0.0%)	0	(0.0%)	14	(27.5%)
Confidence intervals										
Yes	16	(65.2%)^c^	8	(47.1%)	8	(88.9%)	1	(50.0%)	32	(62.7%)
No	8	(34.8%)	9	(52.9%)	1	(11.1%)	1	(50.0%)	19	(37.3%)

^a^The variable for the primary outcome of one meta-analysis is binary, and pooled OR (95% CI) was calculated. However, a person-time-based NNT was calculated by taking the reciprocal of RD between pooled event rates per 1000 patient-years (Preiss 2011)

^b^One study reported relative risk (*RR*) and odds ratio (*OR*) (Maher et al. 2011)

^c^Confidence interval was provided with NNT only for the primary outcome in a study reporting NNT for several outcomes (Green et al. 2007)

### General characteristics of included studies

The majority of studies reporting NNTs were identified from the *Journal of the American Medical Association* (*JAMA*, *n* = 17, 33.3%) and *The Lancet* (*n* = 14, 27.5%) (Additional file 1: Table S7). The median number of papers per year was 5.5 (ranging from 1 in 2009 to 7 in 2011, 2012, and 2014). The included studies were more frequently authored by researchers from the USA (*n* = 21, 41.2%), UK (*n* = 6, 11.8%), and Canada (*n* = 6, 11.8%).

Twenty-three publications (45.1%) were systematic reviews and meta-analyses, while 17 were individual RCTs (33.3%), 9 cohort studies (17.6%), and 2 case–control studies (3.9%). The more frequently studied diseases/conditions were “infections and infestations” (*n* = 7, 13.7%), “cardiac disorders” (*n* = 7, 13.7%), and “psychiatric disorders (*n* = 7, 13.7%).

The primary outcomes of most studies assessed only efficacy (*n* = 30, 58.8%) of interventions. Safety was assessed as the sole primary outcome in 11 studies (21.6%). The remaining 10 studies (19.6%) assessed both efficacy and safety as a primary outcome. The primary outcome was binary in 41 studies (80.4%) and time to event in 10 studies (19.6%).

In addition to NNT estimates, the majority of studies (*n* = 42, 82.4%) also used relative effect measures to express treatment differences. The RR (*n* = 18, 35.3%) and OR (*n* = 16, 31.4%) were the most commonly used.

### Characteristics of NNTs in included studies

NNTs were estimated only for primary outcomes in 28 studies (54.9%), for primary and also secondary outcomes in 21 studies (41.2%), and only for secondary outcomes in 2 studies (3.9%). NNTs were used to assess only benefits of interventions in 26 studies (51.0%), both benefits and harms in 14 studies (27.5%), and only harms in 11 studies (21.6%).

The type of NNT presented in most studies was a person-based NNT (*n* = 40, 78.4%). A person-time-based NNT was presented in 11 studies (21.6%).

The completeness of data presented around the point-estimate NNT was assessed. The baseline risk (i.e., CER) was presented in 37 studies (72.5%), a defined time horizon in 38 studies (74.5%), and CIs in 32 studies (62.7%).

### Assessment of methods used to calculate NNTs

Methods used to calculate NNTs in included studies were compared to basic methodological recommendations (Table 2). A detailed description of data used to assess the completeness of information and the appropriateness of methods used to compute NNTs in included studies is available in Additional file 1: Table S8.

**Table 2:** Assessment of methodology used to calculate number needed to treat (NNT) in included studies

	Meta-analysis (*n* = 23)		RCT (*n* = 17)		Cohort (*n* = 9)		Nested case–control (*n* = 2)		Overall (*n* = 51)	
Methodology used to calculate NNT is defined in the methods section of the study										
Yes	19	82.6%)	0	(0.0%)	7	(77.8%)	2	(100.0%)	28	(54.9%)
No	4	17.4%)	17	(100.0%)	2	(22.2%)	0	(0.0%)	23	(45.1%)
General characteristics of the methodology used to calculate NNT in the study										
Reciprocal of risk difference										
Simple proportions	1	(4.3%)	14	(82.4%)	2	(22.2%)	0	(0.0%)	17	(33.3%)
Cumulative IR	0	(0.0%)	3	(17.6%)	3	(33.3%)	0	(0.0%)	6	(11.8%)
Pooled RD	12	(52.2%)	0	(0.0%)	0	(0.0%)	0	(0.0%)	12	(23.15)
Average RD	0	(0.0%)	0	(0.0%)	4	(44.4%)	0	(0.0%)	4	(7.8%)
Relative effect measure	10	(43.5%)	0	(0.0%)	0	(0.0%)	2	(100.0%)	12	(23.5%)
Methodology used to calculate NNT is in line with basic recommendations (overall)										
Yes	10	(43.5%)	16	(94.1%)	8	(88.9%)	2	(100.0%)	37	(70.6%)
No	13	(56.5%)	1	(5.9%)	1	(11.1%)	0	(0.0%)	15	(29.4%)
Methodology used to calculate NNT is in line with basic recommendations (detailed)										
Binary variables										
Yes	9	(39.1%)	13	(76.5%)	5	(55.6%)	1	(50.0%)	28	(54.9%)
Reciprocal of risk difference										
Simple proportions	0	(0.0%)	13	(76.5%)	1	(11.1%)	0	(0.0%)	14	(27.5%)
Cumulative IR	0	(0.0%)	0	(0.0%)	0	(0.0%)	0	(0.0%)	0	(0.0%)
Pooled RD	0	(0.0%)	0	(0.0%)	0	(0.0%)	0	(0.0%)	0	(0.0%)
Average RD	0	(0.0%)	0	(0.0%)	4	(44.4%)	0	(0.0%)	4	(7.8%)
Relative effect measure	9	(39.1%)	0	(0.0%)	0	(0.0%)	1	(50.0%)	10	(19.6%)
No	13	(56.5%)	0	(0.0%)	0	(0.0%)	0	(0.0%)	13	(25.5%)
Reciprocal of risk difference										
Simple proportions	1	(4.3%)	0	(0.0%)	0	(0.0%)	0	(0.0%)	1	(2.0%)
Cumulative IR	0	(0.0%)	0	(0.0%)	0	(0.0%)	0	(0.0%)	0	(0.0%)
Pooled RD	12	(52.2%)	0	(0.0%)	0	(0.0%)	0	(0.0%)	12	(23.5%)
Average RD	0	(0.0%)	0	(0.0%)	0	(0.0%)	0	(0.0%)	0	(0.0%)
Relative effect measure	0	(0.0%)	0	(0.0%)	0	(0.0%)	0	(0.0%)	0	(0.0%)
Time-to-event variables										
Yes	1	(4.3%)	3	(17.6%)	3	(33.3%)	1	(50.0%)	8	(15.7%)
Reciprocal of risk difference										
Simple proportions	0	(0.0%)	0	(0.0%)	0	(0.0%)	0	(0.0%)	0	(0.0%)
Cumulative IR	0	(0.0%)	3	(17.6%)	3	(33.3%)	0	(0.0%)	6	(11.8%)
Pooled RD	0	(0.0%)	0	(0.0%)	0	(0.0%)	0	(0.0%)	0	(0.0%)
Average RD	0	(0.0%)	0	(0.0%)	0	(0.0%)	0	(0.0%)	0	(0.0%)
Relative effect measure	1	(4.3%)	0	(0.0%)	0	(0.0%)	1	(50.0%)	2	(3.9%)
No	0	(0.0%)	1	(5.9%)	1	(11.1%)	0	(0.0%)	2	(3.9%)
Reciprocal of risk difference										
Simple proportions	0	(0.0%)	1	(5.9%)	1	(11.1%)	0	(0.0%)	2	(3.9%)
Cumulative IR	0	(0.0%)	0	(0.0%)	0	(0.0%)	0	(0.0%)	0	(0.0%)
Pooled RD	0	(0.0%)	0	(0.0%)	0	(0.0%)	0	(0.0%)	0	(0.0%)
Average RD	0	(0.0%)	0	(0.0%)	0	(0.0%)	0	(0.0%)	0	(0.0%)
Relative effect measure	0	(0.0%)	0	(0.0%)	0	(0.0%)	0	(0.0%)	0	(0.0%)

*IR* incidence rate, *RCT* randomized controlled trial, *RD* risk difference

The methodology used to calculate NNT was clearly defined in the methods section of the publications in 28 studies (54.9%). The methodology was not presented in the methods section of the remaining 23 studies (45.1%), but it could be identified using information from other sections of the publications.

Overall, basic methodological recommendations were followed to calculate NNT in 36 studies (70.6%). A summary of the characteristics of studies that did not follow basic methodological recommendations (*n* = 15, 29.4%) is provided in Table 3.

**Table 3:** Characteristics of the included studies in which basic recommendations were not followed to calculate the number needed to treat (NNT)

Study	Variable	Baseline risk	Time horizon	Confidence interval	Methodology used to compute NNT defined in methods section	Method used to compute NNT	Source of data used to compute NNT	Comments
Systematic review and meta-analysis								
Jonas 2014	Binary	No	No	Yes	Yes	NNT = 1/RD	Pooled RD	A pooled RD was calculated for two outcomes. Duration of included trials ranged from 12 to 52 weeks for the outcome any drinking, and from 12 to 24 weeks for heaving drinking
Hempel 2012	Binary	No	No	Yes	Yes	NNT = 1/RD	Pooled RD	The pooled RD (obtained from meta-analysis) led to a loss of follow-up time. Most trials either did not specify the follow-up period, or the assessment was explicitly limited to the time of antibiotics treatment
Leucht 2012	Binary	Yes	Yes	Yes	Yes	NNT = 1/RD	Pooled RD	The outcome is assessed between 7 and 12 months of follow-up; a mean study duration is indicated for each outcome with NNT calculated from absolute RD pooled from the meta-analysis
Shah 2012	Binary	No	No	Yes	Yes	NNT = 1/RD	Pooled RD	The study comprehends the calculation and comparison of NNT for several treatments. However, NNTs are not comparable because they were calculated from pooled RDs and times of follow-up vary considerably across studies included in the meta-analysis (10 days to 48 weeks)
Preiss 2011	Binary	Yes	Yes	No	No	NNT = 1/RD	Pooled RD	The variable for the primary outcome of the study is binary, and pooled OR (95% CI) was calculated. However, NNT was calculated by taking the reciprocal of RD between pooled event rates per 1000 patient-years. Person-time-based NNT was presented and interpreted as the number of persons needed to treat over 1 year
Shamliyan 2011	Binary	Yes	No	Yes	Yes	NNT = 1/RD	Pooled RD	Several antiviral treatments were compared based on estimates of NNT. However, studies with different times of follow-up for antiviral treatments were used to pool absolute RD. The time horizon factor is lost
Coker 2010	Binary	Yes	Yes	Yes	No	NNT = 1/RD	Pooled RD	The pooled RD was obtained for a 14 day follow-up duration in all studies included in the meta-analysis. However, RD varies considerably across the studies included in the meta-analysis (ranging from −8% to 27%)
Testa 2008	Binary	No	No	Yes	Yes	NNT = 1/RD	Pooled RD	Pooled RD was used to calculate NNT. The follow-up of included studies ranged from ”in hospital” to 6 months
Bridge 2007	Binary	Yes	No	Yes	Yes	NNT = 1/RD	Pooled RD	DerSimonian and Laird random-effects model was used to obtain a pooled estimate of the RD (95% CI). NNT was calculated as the reciprocal of RD. The duration of follow-up and the baseline risk varied considerably across included studies
Dentali 2007	Binary	Yes	No	No	Yes	NNT = 1/RD	Simple proportions	Raw totals of patients from each study were added together to estimate proportions and calculate RD, i.e., treating data as if all were from one study (Simpson’s paradox). Further, the baseline risk ranged considerably across included studies (e.g., 0.2–4.0% for pulmonary embolism)
Rovers 2006	Binary	Yes	Yes	No	No	NNT = 1/RD	Pooled RD	Although it is not clearly stated in the methods section, the discussion of the study suggests that the authors calculated pooled RD by means of the meta-analysis
Bongartz 2006	Binary	No	Yes	Yes	Yes	NNT = 1/RD	Pooled RD	NNT calculated for treatment periods of 6–12 months and 3–12 months, using Mantel-Haenszel fixed estimate of absolute RD in cases in which an OR of at least 1.5 was detected
Spiegel 2006	Binary	No	No	No	Yes	NNT = 1/RD	Pooled RD	A pooled RD was calculated for two comparisons. Duration of included trials ranged from 6 to 78 weeks for one comparison and from 12 to 24 weeks for another comparison
Randomized controlled trial								
Shepherd 2008	Time to event	Yes	Yes	No	No	NNT = 1/RD	Simple proportions	NNT calculated as 1/RD using final rates of event and citing a median time of follow-up of 4.8 years (NNT = 14 in patients with diabetes and chronic kidney disease). However, a Kaplan-Meier curve is provided in the study, which should have been used (since the median follow-up is lower than the 5-years objective, at least some patients did not complete the follow-up). From the Kaplan-Meier curve, we would have 20.3% and 14.0% patients with the outcome in the atorvastatin 10 mg and 80 mg/day, respectively, at 4.8 years of follow-up and an NNT = 15.8
Retrospective cohort study								
Graham 2010	Time to event	Yes	Yes	Yes	Yes	NNT = 1/RD	Simple proportions	NNT was calculated using RD between unadjusted incidence rates. Adjusted incidence rates from the Kaplan-Meier curves should have been used. For example, at 1 year of follow-up, NNT for the composite endpoint would be 92 from Kaplan-Meier curves, rather than 60 person-years from unadjusted incidence rates. The authors interpreted person-years as number of persons treated over 1 year, which is not exactly the same

*NNT* number needed to treat, *OR* odds ratio, *RD* risk difference

NNT was calculated as the inverse of the RD between groups in 39 studies (76.5%) (13 meta-analyses, 17 RCTs, and 9 cohort studies). Of those studies, 17 used simple proportions, 12 used pooled RDs, 4 used average RDs, and 6 used cumulative incidence rates. Simple proportions were correctly used in 14 studies (13 RCTs and 1 cohort study) and inappropriately used in 3 studies (1 meta-analysis, 1 RCT, and 1 cohort study). Pooled RDs were always inadequate to the study design (12 meta-analyses). The average RD method was considered to have been correctly used in all 4 studies (4 cohort studies). Cumulative incidence rates were adequately used in all 6 studies (3 cohort studies and 3 RCTs).

The result of a relative effect measure (e.g., OR, RR) was applied to a CER to calculate NNT in 12 studies (23.5%) (10 meta-analyses and 2 case–control studies). The use of this methodology in those studies was in line with basic methodological recommendations.

## Discussion

The present study provides an overview about the use of the NNT in medical research during the last decade. The adherence of selected studies to basic methodological recommendations was reviewed. This topic is particularly relevant given that the NNT concept has been extended to derive related metrics with potential for use in benefit-risk assessments, namely for clinical decision making or drug regulatory purposes. An example is provided by impact numbers, which give a population perspective to the NNT [45, 46]. Impact numbers are useful to describe the public health burden of a disease and the potential impact of a treatment [6]. Two measures of impact numbers are particularly interesting: the number of events prevented in the population (NEPP) and the population impact number of eliminating a risk factor over time $t$ (PIN-ER- $t$) [6, 47, 48].

Clinicians and other investigators should be aware that the calculation and interpretation of NNTs depend on specific study characteristics, particularly the design and outcome variables. The use of inadequate calculating methods may lead to biased results and misleading conclusions [22, 29, 35, 49].

The majority of studies included in the present review aimed at assessing primarily only the efficacy of medical interventions. The NNT was used more often to assess only benefits (51.9%) rather than only harms (21.2%). This finding was expected, considering what is commonly seen in the medical literature. A previous systematic review including meta-analyses published over a 5-year period found that only 14% of studies were designed to investigate drug safety as primary outcome [38]. In another study comprising systematic reviews with absolute effect estimates, it was found that the NNT was mostly used to assess beneficial outcomes rather than harmful events [14].

Overall, included studies reported more frequently results for binary outcomes than for time-to-event outcomes. This finding contrasts with the results of a previous review in which nearly 55% of included studies reported NNTs for time-to-event outcomes [12]. However, that review included only RCTs [12], while the present study included several research designs.

Relative measures of effect were used to express treatment differences in the majority of included studies (82.4%). These findings are in line with the conclusions of a recent survey of 202 systematic reviews [14]. Of those, the majority included meta-analyses with estimation of relative effects (92.1%), while absolute effect estimates were provided in 36.1% [14].

As previously mentioned, the concept of NNT requires the description of a defined period of time and varies with baseline risk (also called CER). Nevertheless, the time horizon was lacking in more than one fourth (25.5%) of studies. The NNT is uninterpretable if the time of follow-up during which cumulative outcome incidences are measured is not provided [34]. In addition, baseline risks could not be ascertained in nearly 28% of studies. Previous findings indicate that 56.2% of studies reporting absolute risks do not present the source of baseline risk estimates [14]. Lastly, more than one third (37.3%) of studies included in the present review did not report the CI for the point-estimate NNT. This result is in line with previous findings [12]. Thus, a moderately high proportion of papers published in journals with high impact factor in the category of “General and/or Internal Medicine” misuse the NNT metric.

As seen across the articles reviewed here, several approaches have been used to derive NNTs from meta-analyses. However, in 13 out of 23 meta-analyses (56.5%) the approach was considered inadequate. Of these meta-analyses, one calculated the reciprocal of simple proportions (using total numbers of both patients with outcome and exposed patients coming from all included studies). Using simple proportions, i.e., treating the data as if they all come from a single trial, to calculate NNTs is not correct, as this method is prone to bias due to Simpson’s paradox [35, 50]. The other 12 meta-analyses inverted pooled RDs, but this method should also be avoided [19, 31, 36, 51]. Absolute RDs are usually not constant and homogeneous across different baseline event rates; therefore, they are rarely appropriate for calculating NNTs from meta-analyses [19, 31, 36, 51]. Moreover, the effects of secular trends on disease risk and time horizon preclude the use of pooled RDs, as they can result in misleading NNTs [36, 51]. Relative effect measures (such as RR and OR) are usually more stable across risk groups than are absolute differences. Thus, pooled estimates of relative effect measures should be used rather than absolute RDs to derive NNTs from meta-analyses [19, 31, 36]. Clinicians should preferably use fixed effects OR, random effects OR or RR, and the patient expected event rate (PEER) to individualize NNT when applying results from meta-analyses in clinical practice [4, 19].

Most RCTs (94.1%) followed basic methodological recommendations to calculate NNTs. It is noteworthy that the majority of included RCTs (13 out of 17) analyzed binary outcomes. Studies with fixed times of follow-up are usually not prone to miscalculation of NNT because cumulative incidences equal simple proportions at the study end [29]. However, previous studies suggested that NNTs are miscalculated in at least half of RCTs with time-to-event outcomes [12, 29].

In the present review, one out four RCTs with varying follow-up times applied a non-recommended method to calculate NNT (see, e.g., [52]). In that RCT, the effect of two doses of atorvastatin (80 mg or 10 mg daily) was tested, for the first occurrence of a major cardiovascular event (i.e., a time-to-event outcome), in patients with coronary artery disease (CAD) and type 2 diabetes, with and without chronic kidney disease [52]. Patients were followed for varying times (median, 4.8 years). Although Kaplan-Meier curves have been estimated, the authors used simple proportions of patients with the outcome to compute NNT (e.g., for patients with diabetes without CAD, 1/([62/441] – [57/444]) = 82) and concluded that 82 patients were needed to treat with 80 mg/day versus 10 mg/day to prevent one major cardiovascular event over 4.8 years [52]. Using the cumulative incidences provided in Kaplan-Meier curves (12.5% for 80 mg and 13.3% for 10 mg), NNT would have been estimated at 125 over the same time horizon. This example illustrates how the use of simple proportions can lead to misleading values of NNT. Simple proportions should be used only if all patients are followed for the entire study period, as they equal cumulative incidences estimated by the Kaplan-Meier approach [30]. Since follow-up times usually vary in RCTs, simple proportions are not valid estimates of cumulative incidences. In cases where follow-up is short and mostly complete, simple proportions and Kaplan-Meier incidences are almost similar [30].

As the present study assessed results from research published since 2006, two different methodologies were considered adequate for calculating NNT from RCTs where the outcome is time to an event [26, 53, 54]. More recently, however, the authors of a study comparing the risk difference approach (reciprocal of risk differences estimated by survival time methods) and the incidence difference approach (reciprocal of incidence rates differences) concluded that the methods based on incidence rates often lead to misleading NNT estimates and recommended the use of survival time methods to estimate NNTs in RCTs with time-to-event outcomes [28]. The incidence difference approach still can be used in the case of small baseline risks, strong treatment effects, and exponentially distributed survival times [28]. Nevertheless, Girerd et al. argued that the two methods measure different things, but both are valid and provide complementary information regarding the absolute effect of an intervention, highlighting that the incidence rate approach assesses person-years rather than persons [55]. This calculating method estimates the number of person-times (e.g. patient-years), not the absolute number of persons, needed to observe one less (or one more) event in the treatment group than in the control group [28, 29, 54–56]. This estimate is different from the “classical” person-based NNT, and therefore may be difficult to interpret [56]. For example, 100 patient-years do not necessarily mean 100 individual patients treated over 1 year (or 50 patients treated for 2 years). A thorough explanation of person-based NNT, person-time-based NNT, and event-based NNT (for multiple recurrent outcome events) is provided elsewhere [29, 57].

With regard to observational studies, one cohort study did not follow methodological recommendations [58]. In that study, Kaplan-Meier curves and Cox proportional HRs for time to event, adjusted for confounding factors, with pioglitazone as reference, were used to test the effect of rosiglitazone on several cardiovascular adverse events [58]. However, the authors applied unadjusted incidence rate differences to calculate NNTs, instead of using adjusted data. For example, at 1 year of follow-up, the NNT for a composite cardiovascular endpoint would be 92 from Kaplan-Meier curves rather than the 60 person-years obtained by the authors. Further, the authors interpreted person-years as number of persons treated over 1 year, which is not exactly the same. A detailed review and discussion of methods used to calculate NNTs from observational studies is provided elsewhere [21–23].

The present study was not primarily aimed at identifying all papers with methodological recommendations for calculating NNTs. For this reason, a systematic review of literature was not performed to identify such papers. This is a potential limitation of the study. Nevertheless, the literature used as the source of evidence was probably adequate for the complexity of the assessment. The study focused on the adherence of calculating methods to basic methodological recommendations, rather than to more complex methodological and statistical issues. Therefore, estimates of NNT reported by studies that followed basic methodological recommendations are not necessarily correct. There are possibly other reasons that can still lead to biased estimates, but which could not be assessed with an acceptable effort. In addition, the magnitude of error produced in studies that did not follow basic methodological recommendations to calculate NNTs was not tested. Aside from some examples provided in the discussion, the calculation of correct NNTs was not sought for studies that did not follow recommendations. Lastly, the study was limited to the top 25 high-impact factor journals in the “General and/or Internal Medicine” category. Whether or not the results in other fields are likely to show similar results deserves further testing.

The present results illustrate that these metrics have not always been adequately calculated. From the clinicians’ point of view, this may cause some concerns, since these metrics can be used to support clinical decision-making processes, including the prescription of medicines. Therefore, clinicians need to rely on the methodological appropriateness of such calculations.

## Conclusions

The NNT helps to quantify the magnitude of effects of medical interventions in an absolute scale, therefore bringing added value to decisions on drug utilization for clinicians, regulators, and other stakeholders. However, they should be aware that the calculation and interpretation of the NNT depend on the characteristics of a given study, namely the design and outcome variables. Moreover, they must acknowledge that an NNT is specific to a given comparison. Therefore, baseline risks, clearly defined outcomes, time horizons, and confidence intervals should be provided. The presentation of an NNT alone, i.e., without its context, would be ambiguous and less useful for decision making.

This study showed that, although the concept of NNT was introduced several years ago, there are basic methodological recommendations still not being followed, particularly in meta-analyses, leading to miscalculated and misinterpreted results. Further research is needed to confirm the present findings and to explore the influence of other methodological aspects that may impact the calculation of the NNT in clinical studies.

## Additional file

Additional file 1: Table S1. List of the 25 journals of “General and/or Internal Medicine” with higher impact factor in 2015. **Table S2.** Search strategy used to identify studies reporting number needed to treat (NNT), performed in PubMed on 24 August 2016. **Table S3.** List of queries used to describe and categorize NNT in selected studies. **Table S4.** List of queries used to assess methodologies used to calculate NNT in selected studies. **Table S5.** Search strategy used to identify studies investigating methods for calculating number needed to treat (NNT), performed in PubMed on 24 August 2016. **Table S6.** Main characteristics of included studies. **Table S7.** Number of publications reporting number needed to treat (NNT) values, according to study design and journal. **Table S8.** Description of data used to assess the completeness of information and the appropriateness of methods used to compute NNTs in the included studies. (DOCX 78 kb)

## Abbreviations

ARR: Absolute risk reduction
BMJ: British Medical Journal
CAD: Coronary artery disease
CER: Control event rate
CI: Confidence interval
CONSORT: Consolidated Standards of Reporting Trials
HR: Hazard ratio
JAMA: Journal of the American Medical Association
MedDRA: Medical Dictionary for Regulatory Activities
NEPP: Number of events prevented in the population
NNE: Number needed to be exposed
NNEB: Number needed to be exposed to benefit
NNEH: Number needed to be exposed to be harmed
NNT: Number needed to treat
NNTB: Number needed to treat to benefit
NNTH: Number needed to treat to be harmed
OR: Odds ratio
PEER: Patient expected event rate
PIN-ER- *t*: Population impact number of eliminating a risk factor over time *t*
RCT: Randomized controlled trial
RD: Risk difference
RR: Relative risk
SOC: System Organ Class
UER: Unexposed event rate
