# Supplementary material for: Number needed to treat (NNT) in clinical literature: an appraisal
Source: BMC Med. 2017 Jun 1;15:112. doi: 10.1186/s12916-017-0875-8 (PMC5455127; doi:10.1186/s12916-017-0875-8)
Supplement: Additional file 1: Table S1. — List of the 25 journals of “General and/or Internal Medicine” with higher impact factor in 2015. Table S2. Search strategy used to identify studies reporting number needed to treat (NNT), performed in PubMed on 24 August 2016. Table S3. List of queries used to describe and categorize NNT in selected studies. Table S4. List of queries used to assess methodologies used to calculate NNT in selected studies. Table S5. Search strategy used to identify studies investigating methods for calculating number needed to treat (NNT), performed in PubMed on 24 August 2016. Table S6. Main characteristics of included studies. Table S7. Number of publications reporting number needed to treat (NNT) values, according to study design and journal. Table S8. Description of data used to assess the completeness of information and the appropriateness of methods used to compute NNTs in the included studies. (DOCX 78 kb) [file 12916_2017_875_MOESM1_ESM.docx]

**Additional file 1**

**Table S1 –** List of the 25 Journals of “General and/or Internal Medicine” with higher Impact Factor in 2015.

| Rank | Full Journal Title | Total Cites | Journal Impact Factor |
| --- | --- | --- | --- |
| 1 | NEW ENGLAND JOURNAL OF MEDICINE | 283,525 | 59.558 |
| 2 | LANCET | 195,553 | 44.002 |
| 3 | JAMA-JOURNAL OF THE AMERICAN MEDICAL ASSOCIATION | 129,909 | 37.684 |
| 4 | BMJ-BRITISH MEDICAL JOURNAL | 93,118 | 19.697 |
| 5 | ANNALS OF INTERNAL MEDICINE | 49,618 | 16.440 |
| 6 | JAMA INTERNAL MEDICINE | 5,590 | 14.000 |
| 7 | PLOS MEDICINE | 20,499 | 13.585 |
| 8 | BMC MEDICINE | 7,331 | 8.005 |
| 9 | JOURNAL OF CACHEXIA SARCOPENIA AND MUSCLE | 901 | 7.883 |
| 10 | JOURNAL OF INTERNAL MEDICINE | 9,090 | 7.803 |
| 11 | CANADIAN MEDICAL ASSOCIATION JOURNAL | 12,420 | 6.724 |
| 12 | MAYO CLINIC PROCEEDINGS | 10,745 | 5.920 |
| 13 | AMERICAN JOURNAL OF MEDICINE | 22,561 | 5.610 |
| 14 | ANNALS OF FAMILY MEDICINE | 3,879 | 5.087 |
| 15 | TRANSLATIONAL RESEARCH | 2,418 | 4.557 |
| 16 | AMERICAN JOURNAL OF PREVENTIVE MEDICINE | 17,735 | 4.465 |
| 17 | ANNALS OF MEDICINE | 4,012 | 3.763 |
| 18 | DEUTSCHES ARZTEBLATT INTERNATIONAL | 2,403 | 3.738 |
| 19 | PALLIATIVE MEDICINE | 3,714 | 3.685 |
| 20 | JOURNAL OF GENERAL INTERNAL MEDICINE | 14,808 | 3.494 |
| 21 | MEDICAL JOURNAL OF AUSTRALIA | 9,739 | 3.369 |
| 22 | AMERICAN JOURNAL OF CHINESE MEDICINE | 2,535 | 2.959 |
| 23 | BRITISH MEDICAL BULLETIN | 3,727 | 2.921 |
| 24 | PREVENTIVE MEDICINE | 12,516 | 2.893 |
| 25 | QJM-AN INTERNATIONAL JOURNAL OF MEDICINE | 5,309 | 2.824 |

Source: InCites^TM^ Journal Citation Reports® by Thomson Reuters.

**Table S2 –** Search strategy used to identify studies reporting number needed to treat (NNT), performed in Pubmed on 24^th^ August 2016.

| **Search** | **Terms** | **Results** |
| --- | --- | --- |
| #1 | Search ((((((((((((((((((((((((("The New England journal of medicine"[Journal]) OR "Lancet (London, England)"[Journal]) OR "JAMA"[Journal]) OR "British medical journal"[Journal]) OR "Annals of internal medicine"[Journal]) OR "JAMA internal medicine"[Journal]) OR "PLoS medicine"[Journal]) OR "BMC medicine"[Journal]) OR ("Journal of cachexia, sarcopenia and muscle"[Journal])) OR "Journal of internal medicine"[Journal]) OR "Canadian Medical Association journal"[Journal]) OR "Mayo Clinic proceedings"[Journal]) OR "The American journal of medicine"[Journal]) OR "Annals of family medicine"[Journal]) OR ("Translational research : the journal of laboratory and clinical medicine"[Journal])) OR "American journal of preventive medicine"[Journal]) OR "Annals of medicine"[Journal]) OR "Deutsches Ärzteblatt international"[Journal]) OR "Palliative medicine"[Journal]) OR "Journal of general internal medicine"[Journal]) OR "The Medical journal of Australia"[Journal]) OR "The American journal of Chinese medicine"[Journal]) OR "British medical bulletin"[Journal]) OR "Preventive medicine"[Journal]) OR "QJM : monthly journal of the Association of Physicians"[Journal]) | 560760 |
| #2 | Search numbers needed to treat[MeSH Terms] | 160 |
| #3 | Search "nnt" | 2333 |
| #4 | Search "nnh" | 639 |
| #5 | Search "nntb" | 216 |
| #6 | Search "nnth" | 110 |
| #7 | Search "number needed to treat"[Title/Abstract] | 3555 |
| #8 | Search "number needed to harm"[Title/Abstract] | 579 |
| #9 | Search ((#2 OR #3 OR #4 OR #5 OR #6 OR #7 OR #8)) | 5040 |
| #10 | Search (#1 AND #9) | 225 |
| #11 | Search ((#1 AND #9)) Sort by: PublicationDate Filters: Publication date from 2006/01/01 to 2015/12/31 | 138 |

**Table S3 –** List of queries used to describe and categorize NNT in selected studies.

| ***Description and categorization of NNT estimates:*** |
| --- |
| 1. What was the type of variable used to compute NNT for the outcome of interest? |
| - Binary; |
| - Time-to-event. |
| 1. Was the NNT presented together with the result of a relative effect measure? |
| - - Yes; |
| - - No. |
| 1. Which was the relative effect measure presented together with the NNT? |
| - - Hazard Ratio; |
| - - Odds Ratio; |
| - - Rate Ratio; |
| - - Relative Risk; |
| - - Not applicable; |
| 1. For which study outcome was the NNT calculated? |
| - - Primary outcome; |
| - - Primary and secondary outcomes; |
| - - Other outcomes than the primary outcome. |
| 1. Was the NNT calculated for beneficial, harmful or both beneficial and harmful outcomes? |
| - - NNTB (beneficial outcome); |
| - - NNTH (harmful outcome); |
| - - NNTB and NNTH. |
| 1. Which type of NNT was calculated? |
| - Patient-based NNT; |
| - Patient-time-based NNT; |
| - Event-based NNT (multiple events). |
| 1. Was the control event rate used to derive the NNT presented? |
| - Yes; |
| - No. |
| 1. Was the time horizon clearly defined for the NNT? |
| - - Yes; |
| - - No. |
| 1. Were the confidence intervals provided for the NNT? |
| - Yes; |
| - No. |

Notes: Patient-based NNT: Number of patients with outcome of interest divided by the total amount of participating patients; Patient-time-based NNT: Number of patients with outcome of interest divided by the total amount of patient-time, to account for varying follow-up times; Event-based NNT (multiple events): Number of outcome events divided by the total amount of patient-time [29].

**Table S4 –** List of queries used to assess methodologies used to calculate NNT in selected studies.

| ***Assessment of the methodology used to calculate NNT:*** |
| --- |
| 1. Was the method used to compute NNT defined in the methods section? |
| - - Yes; |
| - - No; |
| 1. Which method was used to derive the NNT? |
| - - Risk difference (i.e. absolute risk reduction or increase); |
| - - Relative effect measure (e.g., Hazard Ratio; Odds Ratio; Rate Ratio; Relative Risk). |
| 1. What was the source of data used to calculate NNT? |
| - - Simple proportions; |
| - - Relative effect measure; |
| - - Cumulative incidence rates (i.e. using a Kaplan-Meier approach or a Cox regression model); |
| - - Pooled risk differences (i.e. derived from meta-analysis); |
| - - Average risk difference; |
| 1. Was the method used to derive the NNT in line with recommendations for study design? |
| - - Yes; |
| - - No. |

**Table S5 –** Search strategy used to identify studies investigating methods for calculating number needed to treat (NNT), performed in Pubmed on 24^th^ August 2016.

| **Search** | **Terms** | **Results** |
| --- | --- | --- |
| #1 | Search numbers needed to treat[MeSH Terms] | 160 |
| #2 | Search nnt | 2333 |
| #3 | Search nnh | 639 |
| #4 | Search nntb | 216 |
| #5 | Search nnth | 110 |
| #6 | Search "number needed to treat"[Title/Abstract] | 3555 |
| #7 | Search "number needed to harm" | 579 |
| #8 | Search (#1 OR #2 OR #3 OR #4 OR #5 OR #6 OR #7) | 5040 |
| #9 | Search ("Epidemiologic Methods"[Majr] OR "Data Collection"[Majr] OR "Data Interpretation, Statistical"[Majr] OR "Statistics as Topic"[Majr] OR "Evidence-Based Medicine"[Mesh]) | 619049 |
| #10 | Search (#8 AND #9) | 629 |

**Table S6 –** Main characteristics of included studies.

| Reference | Journal | Year | Country | Studies, N= | Participants, N= | Study duration | Disease or condition | Disease, SOC | Intervention | Control | Primary outcome measure |
| --- | --- | --- | --- | --- | --- | --- | --- | --- | --- | --- | --- |
| Systematic review and Meta-analysis | | | | | | | | | | | |
| Palmerini et al. | Lancet | 2015 | Italy | 10 | 31,666 | NA | Patients with CAD undergoing PCI | Cardiac disorders | Short-duration DAPT | Long-duration (>1 yr) DAPT | All-cause mortality |
| Chatterjee et al. | JAMA | 2014 | USA | 16 | 2,115 | NA | Pulmonary embolism | Respiratory, thoracic and mediastinal disorders | Thrombolytic therapy | Conventional anticoagulant therapy | All-cause mortality and major bleeding |
| Jonas et al. | JAMA | 2014 | USA | 123* | 22,803 | NA | Alcoholism | Psychiatric disorders | Medication for alcoholism disorders | Placebo or other medication | Not clearly defined. Several outcomes analysed: alcohol consumption, motor vehicle crashes, injuries, quality of life, function, mortality, and harms |
| Spielmans et al. | PLoS Med | 2013 | USA | 7 | 3,549 | NA | Depression | Psychiatric disorders | Atypical antipsychotics | Placebo | A primary outcome is not clearly defined. Several outcomes analysed: remission, response, and adverse events. |
| Kayentao et al. | JAMA | 2013 | UK | 7 | 6,281 | NA | Pregnancy | Pregnancy, puerperium and perinatal conditions | 3 or more doses of sulfadoxine-pyrimethamine | 2 doses of sulfadoxine-pyrimethamine | Low birth weight (LBW); and mean birth weight |
| Hempel et al. | JAMA | 2012 | USA | 82 | 11,811 | NA | Antibiotic-associated diarrhea | Gastrointestinal disorders | Probiotics (Lactobacillus, Bifidobacterium, Saccharomyces, Streptococcus, Enterococcus, and/orBacillus) | No treatment, placebo, or a different probiotic or probiotic dose | Participants with diarrhea |
| Leucht et al. | Lancet | 2012 | Germany | 65 | 6,493 | NA | Schizophrenia | Psychiatric disorders | Antipsychotics | Placebo | Relapse between 7 and 12 months |
| Shah et al. | Am J Med | 2012 | USA | 18 | 8,595 | NA | Irritable bowel syndrome | Gastrointestinal disorders | Alosetron; tricyclic antidepressants; rifaximin; lubiprostone; or selective serotonin reuptake inhibitors | Placebo | Adverse event requiring discontinuation of treatment |
| Maher et al. | JAMA | 2011 | USA | 393 |  | NA | Off-label conditions (agitation in dementia, anxiety, and OCD) | Injury, poisoning and procedural complications | Atypical antipsychotics (risperidone, olanzapine, quetiapine, aripiprazole, ziprasidone, asenapine, iloperidone, or paliperidone) | Placebo, another atypical antipsychotic medication, or other pharmacotherapy for adult off-label conditions | A primary outcome is not clearly defined. Several outcomes analysed: improvement in psychosis, improvement in agitation, and a total global score. |
| Preiss et al. | JAMA | 2011 | UK | 5 | 32,752 | NA | Cardiovascular disease | Cardiac disorders | Intensive-dose statin therapy | Moderate-dose statin therapy | Major cardiovascular events (prevention); New-onset diabetes (adverse event) |
| Shamliyan et al. | J Gen Intern Med | 2011 | USA | 16 | 4,431 | NA | Chronic hepatitis B | Infections and infestations | Antiviral drugs | Antiviral drugs or placebo | A primary outcome is not clearly defined. Several outcomes analysed: mortality, incidence of hepatocellular carcinoma, liver failure, prevalence and incidence of cirrhosis, HBeAg or HBsAg presence or seroconversion, viral load of HBV deoxyribonucleotide acid (HBV DNA), ALT levels, histological necroinflammatory and fibrosis scores, and adverse events. |
| Coker et al. | JAMA | 2010 | USA | 7 | 2,058 | NA | Acute Otitis Media | Infections and infestations | Antibiotics (immediate initiation) | Placebo or strategy of observation with possible delayed treatment | Clinical success |
| Testa et al. | Q J Med | 2008 | UK | 8 | 1,318 | NA | Myocardial infarction | Cardiac disorders | Rescue PCI or repeat thrombolysis | Conservative therapy | A primary outcome is not clearly defined. Several outcomes analysed: major adverse events, defined as the composite of overall mortality and re-infarction; stroke, congestive heart failure (CHF), major bleeds, and minor bleeds |
| Bangalore et al. | Lancet | 2008 | USA | 33 | 12,306 | NA | Non-cardiac surgery | Surgical and medical procedures | β blockers (intravenous or oral) | Other drugs, placebo, or no intervention | A primary outcome is not clearly defined. Several outcomes analysed: 30-day all-cause mortality, cardiovascular mortality, non-fatal myocardial infarction, non-fatal stroke, and heart failure; perioperative adverse events (bradycardia, hypotension, and bronchospasm) |
| Christensen et al. | Lancet | 2007 | Denmark | 4 | 4,105 | NA | Obesity | Metabolism and nutrition disorders | Rimonabant 20 mg | Placebo | Difference in mean weight change and the number of individuals achieving at least 10% weight reduction handled as a dichotomous responder criterion |
| Green et al. | Mayo Clin Proc | 2007 | Israel | 12 | 1,245 | NA | Pneumocystis pneumonia (PCP) prophylaxis (caused by Pneumocystis jirovecii) | Infections and infestations | Antibiotics | Placebo, no intervention, or antibiotics with no activity against P jirovecii | PCP infection |
| Bridge et al. | JAMA | 2007 | USA | 27 | 5,31 | NA | Major depressive disorder (MDD), OCD, and non-OCD anxiety disorders | Psychiatric disorders | SSRIs and other second generation antidepressants | Placebo | Efficacy: treatment response and the prospectively identified scalar variable assessing change in symptoms from baseline to the end of treatment; safety: suicidal ideation/suicide attempt |
| Leontiadis et al. | Mayo Clin Proc | 2007 | USA | 24 | 4,373 | NA | Peptic ulcer bleeding | Gastrointestinal disorders | Proton pump inhibitors (PPIs) | Placebo or a histamine 2-receptor antagonist | Mortality from any cause within 30 days of randomization |
| Dentali et al. | Ann Intern Med | 2007 | Canada | 9 | 19,958 | NA | Hospitalized medical patients at risk for venous thromboembolism | Vascular disorders | Anticoagulant prophylaxis | No anticoagulant prophylaxis | A primary outcome is not clearly defined. Several outcomes analysed: any pulmonary embolism (PE), fatal PE, symptomatic deep venous thrombosis (DVT), and all-cause mortality; and major bleeding |
| Rovers et al. | Lancet | 2006 | The Netherlands | 6 | 1,643 | NA | Acute media otitis | Infections and infestations | Antibiotics (immediate initiation) | Placebo (or delayed treatment with antibiotics) | Extended course of acute otitis media, consisting of pain, fever, or both at 3–7 days |
| Hollingsworth et al. | Lancet | 2006 | USA | 9 | 693 | NA | Urinary stone disease | Renal and urinary disorders | Calcium-channel blockers or α-blockers | Non-use of calcium-channel blockers or α-blockers | Proportion of patients who passed stones (cumulative incidence) |
| Bongartz et al. | JAMA | 2006 | UK | 9 | 41,005 | NA | Rheumatoid arthritis | Musculoskeletal and connective tissue disorders | Anti-TNF therapy | Placebo | Serious infections and malignancies |
| Spiegel et al. | Am J Med | 2006 | USA | 26 | 41,529 | NA | Chronic arthritis pain | Musculoskeletal and connective tissue disorders | COX-2 inhibitors or NSAIDs + PPIs | NSAIDs | Dyspepsia |
| Randomized Controlled Trial | | | | | | | | | | | |
| Lenze et al. | Lancet | 2015 | Canada | NA | 181 | 12 weeks | Depression | Psychiatric disorders | Aripiprazole | Placebo | Remission of depression |
| Unger et al. | BMC Medicine | 2015 | Australia | NA | 2,793 | Until delivery | Pregnancy | Pregnancy, puerperium and perinatal conditions | Sulphadoxine-pyrimethamine plus azithromycin | Sulphadoxine-pyrimethamine and chloroquine plus placebo | Proportion of live born, singleton infants without congenital malformations with low birthweight |
| Imazio et al. | JAMA | 2014 | Italy | NA | 360 | 3 months | Patients undergoing cardiac surgery | Surgical and medical procedures | Colchicine | Placebo | Occurrence of postpericardiotomy syndrome |
| Imazio et al. | Lancet | 2014 | Italy | NA | 240 | 18 months | Recurrent pericarditis | Cardiac disorders | Colchicine | Placebo | Recurrence of pericarditis |
| Lazzerini et al. | JAMA | 2013 | Italy | NA | 54 | 8 weeks | Refractory paediatric Crohn’s disease | Gastrointestinal disorders | Thalidomide | Placebo | Clinical remission; Reduction in Pediatric Crohn Disease Activity Index (PCDAI) score by 25% or 75% |
| Mason et al. | JAMA Intern Med | 2014 | USA | NA | 150 | 12 weeks | Alcoholism | Psychiatric disorders | Gabapentin | Placebo | Complete abstinence; no heavy drinking |
| Liou et al. | Lancet | 2013 | Taiwan | NA | 900 | 7 weeks | H pylori infection | Infections and infestations | S14 | T14 | Eradication rate in first-line treatment |
| Enden et al. | Lancet | 2012 | Norway | NA | 209 | 24 months | Deep vein thrombosis | Vascular disorders | Catheter-directed thrombolysis (CDT) using alteplase + Conventional treatment | Conventional treatment with initial low molecular weight heparin (LMWH) and warfarin followed by warfarin alone | Iliofemoral patency after 6 months; and frequency of post-thrombotic syndrome (PTS) after 24 months |
| Ryan et al. | Lancet | 2012 | Australia | NA | 62 | 8 weeks | Refractory chronic cough | Respiratory, thoracic and mediastinal disorders | Gabapentin | Placebo | Change in cough-specifi c quality of life (Leicester cough questionnaire [LCQ] score) from baseline to 8 weeks of treatment |
| Srinivasan et al. | BMC Medicine | 2012 | Uganda | NA | 352 | 7 days‡ | Pneumonia | Infections and infestations | Zinc | Placebo | Time taken for normalization of respiratory rate, time taken for normalization of temperature and time taken for oxygen saturation to normalize (92% or more), while breathing room air |
| Franklin et al. | JAMA | 2011 | USA | NA | 124 | 12 weeks | Pediatric OCD | Psychiatric disorders | Medication management plus instructions in cognitive behaviour therapy (CBT); medication management plus CBT | Medication management only | Proportion of patients responding positively to treatment by improving their baseline obsessive-compulsive scale score by 30% or more |
| Imazio et al. | Ann Intern Med | 2011 | Italy | NA | 120 | 18 months | Recurrent pericarditis | Cardiac disorders | Colchicine | Placebo | Recurrence rate of pericarditis |
| Brinks et al. | Ann Fam Med | 2011 | The Netherlands | NA | 120 | 12 months | Greater trochanteric pain syndrome (GTPS) | Musculoskeletal and connective tissue disorders | Usual care and local corticosteroid injection | Usual care | Recovery at 3 and 12 months as measured on a 7-point Likert Scalre, and severity of pain during last week measured with a numeric rating scale |
| Zinman et al. | Lancet | 2010 | Canada | NA | 207 | 3.9 years | Impaired glucose tolerance | Metabolism and nutrition disorders | Rosiglitazone + Metformin | Placebo | Development of new onset type 2 diabetes |
| Kenyon et al. | Lancet | 2008 | UK | NA | 3,196 | 7 years | Children at age 7 years born to the women who had completed the ORACLE II Study | Pregnancy, puerperium and perinatal conditions | Erythromycin and/or amoxicillin–clavulanate | Placebo | Any level of functional impairment |
| Shepherd et al. | Mayo Clin Proc | 2008 | UK | NA | 10,001 | 4.8 years | CAD, Diabetes, and Chronic Kidney Disease | Cardiac disorders | High-dose atorvastatin | Low-dose atorvastatin | Major cardiovascular events |
| Halonen et al. | JAMA | 2007 | Finland | NA | 241 | 84 hours | Cardiac surgery (prevention of atrial fibrillation) | Surgical and medical procedures | Hydrocortisone | Placebo | Atrial fibrillation |
| Retrospective Cohort Study | | | | | | | | | | | |
| Jørgensen et al. | JAMA Intern Med | 2015 | Denmark | NA | 55,32 | 30 days | Hypertension | Vascular disorders | Beta-blocker | Other antihypertensive drugs | MACE and all-cause mortality |
| Smith et al. | Am J Med | 2015 | USA | NA | 11,18 | 1 year | Myocardial infarction | Cardiac disorders | Statin | Non-use of statin | All-cause mortality and CV hospitalizations; and AEs (diabetes mellitus and myopathy) |
| Parekh et al. | JAMA Intern Med | 2014 | USA | NA | 30,411 | 14 days | Diabetes and concomitant infection | Metabolism and nutrition disorders | Antimicrobials known to cause hypoglycaemia | Antimicrobials not linked to hypoglycemia | Any hospitalization or emergency department visit owing to hypoglycemia within 14 days of antimicrobial exposure |
| Fexer et al. | Dtsch Arztebl Int | 2014 | Germany | NA | 2,992 | 42 months | COPD | Respiratory, thoracic and mediastinal disorders | Teophyline | Non-use of theophyline | Hospitalizations and disease exacerbations |
| London et al. | JAMA | 2013 | USA | NA | 55,138 | 30 days | Noncardiac surgery | Surgical and medical procedures | Beta-blocker | Non-use of beta-blocker | All-cause mortality |
| Meropol et al. | Ann Fam Med | 2013 | USA | NA | 814,283 | 15 days | Acute nonspecific respiratory infections | Infections and infestations | Antibiotics | Non-use of antibiotics | Hospitalization within 15 days for severe adverse drug events and community-acquired pneumonia |
| Leung et al. | Am J Med | 2011 | USA | NA | 2,613 | 10 years† | Hypertension | Vascular disorders | Thiazide | Non-use of thiazide | First occurrence of hyponatremia |
| Graham et al. | JAMA | 2010 | USA | NA | 227,571 | Until the earliest occurrence of a study end point | Type 2 diabetes mellitus | Metabolism and nutrition disorders | Rosiglitazone | Pioglitazone | Individual end points of acute myocardial infarction (AMI), stroke, heart failure, and all-cause death, and composite end point of AMI, stroke, heart failure, or death |
| Wijeysundera et al. | Lancet | 2008 | Canada | NA | 259,037 | 30 days | Elective surgical procedures | Surgical and medical procedures | Epidural anaesthesia | No epidural anaesthesia | All-cause death within 30 days after surgery |
| Nested case-control | | | | | | | | | | | |
| Etminan et al. | JAMA | 2012 | Canada | NA | 989,591 | 1,7 years | Ophtalmological condition | Eye disorders | Oral fluoroquinolones | Non-use of fluoroquinolones | Retinal detachment |
| Bell et al. | JAMA | 2009 | Canada | NA | 96,128 | 14 days | Cataract | Eye disorders | Tansulosin or other alfa-blocking drugs | No exposure in the year prior to cataract surgery | Postoperative ophthalmic adverse events (a composite of procedures signifying retinal detachment, lost lens or lens fragment, or endophthalmitis occurring within 14 days after cataract surgery) |

**Legend:**

AE, adverse event; AMI, acute myocardial infarction; CAD, coronary artery disease; CER, control event rate; CHF, congestive heart failure; COPD, Chronic Obstructive Pulmonary Disease; CV, cardiovascular; DAPT, dual antiplatelet therapy; DVT, deep venous thrombosis; ICU: Intensive Care Unit; MACE, major adverse cardiovascular event; mo, months; MedDRA Medical Dictionary for Regulatory Activities; NA, not applicable; NNT, number needed to treat; NNTB, number needed to treat to benefit; NNTH, number needed to treat to harm; NSAIDs, non-steroidal anti-inflammatory drugs; OCD, obsessive-compulsive disorder; PCI, percutaneous coronary intervention; PCP, Pneumocystis pneumonia; PE, pulmonary embolism; PPIs, proton-pump inhibitors; RD, risk difference; SOC, system organ class; SSRIs, selective serotonin reuptake inhibitors; TNF, tumor necrosis factor; wk, weeks; yr, years;

*122 RCT and 1 cohort study;

‡ Until hospital discharge, death or a maximum of 7 days, whichever came first;

† Subjects were followed from their index date until first occurrence of hyponatremia, death, or December 31, 2009 (whichever came first), providing for a maximum follow-up of 10 years;

¥ Extension (follow-up) of a RCT; ORACLE II: The ORACLE II trial compared the use of erythromycin and/or amoxicillin–clavulanate (co-amoxiclav) with that of placebo for women in spontaneous preterm labour and intact membranes, without overt signs of clinical infection, by use of a factorial randomised design;

₹ Median follow-up before co-trimoxazole was 154 (IQR 147–161) days, during co-trimoxazole was 532 (488–542) days, and during ART and co-trimoxazole was 749 (699–812) days.

**Table S7 –** Number of publications reporting number needed to treat (NNT) values, according to study design and journal.

| **Journal** | **Study Design** | | | | **Total** |
| --- | --- | --- | --- | --- | --- |
|  | **Systematic review and meta-analysis** | **RCT** | **Cohort** | **Case-control** |  |
| Am J Med | 2 | 0 | 2 | 0 | 4 |
| Ann Fam Med | 0 | 1 | 1 | 0 | 2 |
| Ann Intern Med | 1 | 1 | 0 | 0 | 2 |
| BMC Medicine | 0 | 2 | 0 | 0 | 2 |
| Dtsch Arztebl Int | 0 | 0 | 1 | 0 | 1 |
| J Gen Intern Med | 1 | 0 | 0 | 0 | 1 |
| JAMA | 9 | 4 | 2 | 2 | 17 |
| JAMA Intern Med | 0 | 1 | 2 | 0 | 3 |
| Lancet | 6 | 7 | 1 | 0 | 14 |
| Mayo Clin Proc | 2 | 1 | 0 | 0 | 3 |
| PLoS Med | 1 | 0 | 0 | 0 | 1 |
| QJM | 1 | 0 | 0 | 0 | 1 |
| Total | 23 | 17 | 9 | 2 | 51 |

Legend: RCT, Randomized controlled trial.

**Table S8 –** Description of data used to assess the completeness of information and the appropriateness of methods used to compute NNTs in the included studies.

| Reference | Primary outcome type | Type of variable | Relative effect measure | Type of relative effect measure | NNT calculated for outcome | NNTB and/or NNTH | Type of NNT | CER presented | Time horizon defined for NNT | Confidence intervals for NNT | Methodology used to compute NNT defined in methods section | Method used to compute NNT | Source of data used to compute NNT | Adequate method to compute NNT | Comments |
| --- | --- | --- | --- | --- | --- | --- | --- | --- | --- | --- | --- | --- | --- | --- | --- |
| Systematic review and Meta-analysis | | | | | | | | | | | | | | | |
| Palmerini et al. | Efficacy | Time to event | Yes | Hazard Ratio | Primary and secondary outcomes | NNTB | Patient-time-based NNT | No | Yes | No | Yes | Relative effect measure | Relative effect measure | Yes | NNT calculated using ‘metannt’ command with STATA. The RD is calculated based on an assumed value of the risk in the control group. The ‘metannt’ command calculates this by deriving an estimate of the intervention effect (e.g. a risk ratio), applying it to a population with a given outcome event risk, and deriving from this a projected event risk if the population were to receive the intervention. |
| Chatterjee et al. | Efficacy and Safety | Binary | Yes | Odds ratio | Primary and secondary outcomes | NNTB; NNTH | Patient-based NNT | Yes | No | Yes | Yes | Relative effect measure | Relative effect measure | Yes* | *The method used to compute NNT is adequate. However, the CER was obtained using the total number of events and total patients from all studies in the meta-analysis (Simpsons' paradox). |
| Jonas et al. | Efficacy and Safety | Binary | No | NA | Primary and secondary outcomes | NNTB; NNTH | Patient-based NNT | No | No | Yes | Yes | NNT=1/RD | Pooled RD | No | NNTB and NNTH were calculated only when pooled RDs found a statistically significant result. A pooled RD was calculated for two outcomes. Duration of included trials ranged from 12 to 52 weeks for the outcome any drinking, and from 12 to 24 weeks for heaving drinking. |
| Spielmans et al. | Efficacy and Safety | Binary | Yes | Odds ratio | Primary and secondary outcomes | NNTB; NNTH | Patient-based NNT | No | No | Yes | Yes | Relative effect measure | Relative effect measure | Yes | Although not presented, authors stated that "The baseline risk was calculated separately for each drug, so that placebo participants in one drug’s trials were not used to calculate baseline risk for a different drug". Conversions from OR to NNT were performed using Visual Rx software. |
| Kayentao et al. | Efficacy | Binary | Yes | Relative risk | Primary outcome | NNTB | Patient-based NNT | Yes | Yes | No | Yes | Relative effect measure | Relative effect measure | Yes | Time horizon considered well defined because the outcome is assessed at delivery (after pregnancy). |
| Hempel et al. | Efficacy | Binary | Yes | Relative risk | Primary outcome | NNTB | Patient-based NNT | No | No | Yes | Yes | NNT=1/RD | Pooled RD | No | The pooled risk difference (obtained from meta-analysis) lead to a loss of follow-up time (indeed "Most trials either did not specify the follow-up period, or the assessment was explicitly limited to the time of antibiotics treatment") |
| Leucht et al. | Efficacy | Binary | Yes | Relative risk | Primary and secondary outcomes | NNTB; NNTH | Patient-based NNT | Yes | Yes | Yes | Yes | NNT=1/RD | Pooled RD | No | The outcome is assessed between 7 and 12 months of follow-up; a mean study duration is indicated for each outcome with NNT (calculated from absolute RD pooled from the meta-analysis). |
| Shah et al. | Safety | Binary | Yes | Relative Risk | Primary and secondary outcomes | NNTB; NNTH | Patient-based NNT | No | No | Yes | Yes | NNT=1/RD | Pooled RD | No | The study comprehends the calculation and comparison of NNT for several treatments. However, NNTs are not comparable because they were calculated from pooled RDs and times of follow-up vary considerably across studies included in the meta-analysis (10 days to 48 weeks). |
| Maher et al. | Efficacy and Safety | Binary | Yes | Relative risk; Odds Ratio | Primary and secondary outcomes | NNTH | Patient-based NNT | No | No | No | Yes | Relative effect measure | Relative effect measure | Yes* | NNT was calculated for significant RRs (NNTB) or ORs (NNTH) using pooled RR and the assumed control risk from the placebo group). *The method is adequate; however, the authors did not clearly define the time-horizon and did not provide the control event rate used to compute NNTs. |
| Preiss et al. | Efficacy and Safety | Binary | Yes | Odds ratio | Primary outcome | NNTB; NNTH | Patient-time-based NNT | Yes | Yes | No | No | NNT=1/RD | Pooled RD | No | The variable for the primary outcome of the study is binary and pooled OR (95% CI) was calculated. However, NNT was calculated by taking the reciprocal of RD between pooled event rates per 1000 patient-years. Person-time based NNT was presented and interpreted as the number of persons needed to treat over one year. |
| Shamliyan et al. | Efficacy | Binary | No | NA | Primary and secondary outcomes | NNTB | Patient-based NNT | Yes | No | Yes | Yes | NNT=1/RD | Pooled RD | No | Several antiviral treatments were compared based on estimates NNT. However, studies with different times of follow-up for antiviral treatments were used to pool absolute RD. The time horizon factor is lost. |
| Coker et al. | Efficacy | Binary | No | NA | Primary and secondary outcomes | NNTB; NNTH | Patient-based NNT | Yes | Yes | Yes | No | NNT=1/RD | Pooled RD | No | The pooled RD was obtained for a 14 day follow-up duration in every studies included in the meta-analysis. However, RD varies considerably across the studies included in the meta-analysis (ranging from -8% to 27%). |
| Testa et al. | Efficacy and Safety | Binary | Yes | Odds ratio | Primary and secondary outcomes | NNTB; NNTH | Patient-based NNT | No | No | Yes | Yes | NNT=1/RD | Pooled RD | No | Pooled RD was used to calculate NNT. The follow-up of included studies ranged from ‘in hospital’ to 6 months. |
| Bangalore et al. | Efficacy and Safety | Binary | Yes | Odds ratio | Primary and secondary outcomes | NNTB; NNTH | Patient-based NNT | No | Yes | No | Yes | Relative effect measure | Relative effect measure | Yes | Time-horizon was 30 days within the surgery. CERs used to compute NNTs were not presented. Although the methodology used to compute NNT was cited in the methods section, the formula was not provided. |
| Christensen et al. | Efficacy | Binary | Yes | Odds ratio | Primary and secondary outcomes | NNTB; NNTH | Patient-based NNT | Yes | Yes | Yes | Yes | Relative effect measure | Relative effect measure | Yes | On the basis of combined OR values, NNTB and NNTH applying the overall event rate in the placebo group as a proxy for baseline risk. |
| Green et al. | Efficacy | Binary | Yes | Relative Risk | Primary and secondary outcomes | NNTB; NNTH | Patient-based NNT | Yes | No | Yes* | Yes | Relative effect measure | Relative effect measure | Yes | *Confidence interval for the NNT was provided for the primary outcome within the primary analysis. Other NNTs were estimated according to different CERs, but without confidence intervals. |
| Bridge et al. | Efficacy and Safety | Binary | Yes | Relative Risk | Primary and secondary outcomes | NNTB; NNTH | Patient-based NNT | Yes | No | Yes | Yes | NNT=1/RD | Pooled RD | No | DerSimonian and Laird random-effects model was used to obtain a pooled estimate of the RD (95% CI). NNT was calculated as the reciprocal of RD. The duration of follow-up and the baseline risk varied considerably across included studies. |
| Leontiadis et al. | Efficacy | Binary | Yes | Odds ratio | Primary and secondary outcomes | NNTB | Patient-based NNT | Yes | Yes | Yes | No | Relative effect measure | Relative effect measure | Yes | NNTs were estimated using OR and unweighted pooled rates in control groups. The methodology used to compute NNT is considered correct, but it is not clearly described in methods section. |
| Dentali et al. | Efficacy and Safety | Binary | Yes | Relative Risk | Primary and secondary outcomes | NNTB; NNTH | Patient-based NNT | Yes | No | No | Yes | NNT=1/RD | Simple proportions | No | Raw totals of patients from each study were added together to estimate proportions and calculate RD, i.e. treating data as it all came from one study (Simpson’s paradox). Further, the baseline risk ranged considerably across included studies (e.g. 0.2% to 4.0% for pulmonary embolism). |
| Rovers et al. | Efficacy | Binary | Yes | Relative Risk | Primary and secondary outcomes | NNTB | Patient-based NNT | Yes | Yes | No | No | NNT=1/RD | Pooled RD | No | Although it is not clearly stated in the methods section, the discussion of the study suggest that the authors calculated pooled RD by means of the meta-analysis. |
| Hollingsworth et al. | Efficacy | Binary | Yes | Relative Risk | Primary outcome | NNTB | Patient-based NNT | Yes | No | Yes | Yes | Relative effect measure | Relative effect measure | Yes | Several CERs were indicated and used to calculate several NNT from risk ratios (as a relative risk) according to different baseline risks. CERs were not associated with a time-horizon. |
| Bongartz et al. | Safety | Binary | Yes | Odds ratio | Primary outcome | NNTH | Patient-based NNT | No | Yes | Yes | Yes | NNT=1/RD | Pooled RD | No | NNT calculated for treatment periods of 6 to 12 months and 3 to 12 months, using Mantel-Haenszel fixed-estimate of absolute RD in cases in which an OR of at least 1.5 was detected |
| Spiegel et al. | Efficacy | Binary | Yes | Relative Risk | Primary outcome | NNTB | Patient-based NNT | No | No | No | Yes | NNT=1/RD | Pooled RD | No | A pooled RD was calculated for two comparisons. Duration of included trials ranged from 6 to 78 weeks for one comparison; and from 12 to 24 weeks for another comparison. |
| Randomized Controlled Trial | | | | | | | | | | | | | | | |
| Lenze et al. | Efficacy | Binary | Yes | Odds ratio | Primary outcome | NNTB | Patient-based NNT | Yes | Yes | Yes | No | NNT=1/RD | Simple proportions | Yes | All patients completed the follow-up period. |
| Unger et al. | Efficacy | Binary | Yes | Relative Risk | Primary outcome | NNTB | Patient-based NNT | Yes | Yes | No | No | NNT=1/RD | Simple proportions | Yes | Only women with birth outcome was considered for the analysis, so there is no loss of follow-up. |
| Imazio et al. | Efficacy | Binary | No | NA | Primary and secondary outcomes | NNTB; NNTH | Patient-based NNT | Yes | Yes | No | No | NNT=1/RD | Simple proportions | Yes | No patients were lost to follow-up and all were analyzed for outcomes. Absolute differences were provided, but not relative effect measures. |
| Imazio et al. | Efficacy | Binary | Yes | Relative risk | Primary outcome | NNTB | Patient-based NNT | Yes | Yes | No | No | NNT=1/RD | Simple proportions | Yes | No patients were lost to follow-up. |
| Lazzerini et al. | Efficacy | Binary | Yes | Relative Risk | Primary outcome | NNTB | Patient-based NNT | Yes | Yes | Yes | No | NNT=1/RD | Simple proportions | Yes* | *Assuming that no patient was lost to follow-up since all randomized patients were considered in all calculations. |
| Mason et al. | Efficacy | Binary | Yes | Odds ratio | Primary outcome | NNTB | Patient-based NNT | Yes | Yes | Yes | No | NNT=1/RD | Simple proportions | Yes | Although only 57% of patients completed the study (85/150), we considered the method appropriate for computing NNT results since the authors of the study managed to classify all patients as responders or non-responders, including those that left the study earlier. |
| Liou et al. | Efficacy | Binary | No | NA | Primary outcome | NNTB | Patient-based NNT | Yes | Yes | Yes | No | NNT=1/RD | Simple proportions | Yes | About 5% loss at follow-up; patients who did not return for a follow-up 13C-UBT were recorded as treatment failure. |
| Enden et al. | Efficacy | Binary | No | NA | Primary outcome | NNTB | Patient-based NNT | Yes | Yes | Yes | No | NNT=1/RD | Simple proportions | Yes | Only patients completing follow-up were assessed. |
| Ryan et al. | Efficacy | Binary | No | NA | Primary outcome | NNTB | Patient-based NNT | Yes | Yes | No | No | NNT=1/RD | Simple proportions | Yes | Only patients completing follow-up were assessed. Primary outcome was assessed after dichotomization of a continuous variable (mean change). |
| Srinivasan et al. | Efficacy | Binary | Yes | Relative risk | Secondary outcome | NNTB | Patient-based NNT | Yes | Yes | No | No | NNT=1/RD | Simple proportions | Yes | All participants completed the follow-up. However the follow-up for those dying before the 7 days was shorter. |
| Franklin et al. | Efficacy | Binary | No | NA | Primary outcome | NNTB | Patient-based NNT | Yes | Yes | No | No | NNT=1/RD | Simple proportions | Yes* | A total of 81.5% of patients completed the 12 weeks of follow-up. *As part of the study design, efforts were made to collect all outcomes on all randomized participants even when treatment was prematurely terminated. Prior to analysis, multiple imputation was used to replace missing values. A sequential regression multivariate imputation algorithm was used, as implemented in the IVEware package for SAS. |
| Imazio et al. | Efficacy | Time to event | Yes | Relative risk (reduction) | Primary outcome | NNTB | Patient-time-based NNT | Yes | Yes | Yes | No | NNT=1/RD | Cumulative incidence rates | Yes | No patient was lost to follow-up, and all participants were analyzed for outcomes according to their original assigned groups. The recurrence rate was calculated using Cox regression analysis. |
| Brinks et al. | Efficacy | Binary | Yes | Odds ratio | Primary outcome | NNTB | Patient-based NNT | Yes | Yes | No | No | NNT=1/RD | Simple proportions | Yes | NNT for 3 months of follow-up. Only one patient was lost to follow-up at 3 months. |
| Zinman et al. | Efficacy | Time to event | Yes | Hazard ratio | Primary outcome | NNTB | Patient-time-based NNT | Yes | Yes | Yes | No | NNT=1/RD | Cumulative incidence rates | Yes | Separate product-limit estimated cumulative incidence curves were calculated for the two treatment groups and compared with the log-rank test. Cox proportional hazards models were used to assess the effect of rosiglitazone and metformin on the hazard of the primary outcome. |
| Kenyon et al. | Safety | Binary | Yes | Odds ratio | Secondary outcome | NNTH | Patient-based NNT | Yes | Yes | Yes | No | NNT=1/RD | Simple proportions | Yes | NNT calculated for a secondary outcome from proportion of children with cerebral palsy whose mothers had participated in ORACLE II Study |
| Shepherd et al. | Efficacy | Time to event | Yes | Hazard ratio | Primary outcome | NNTB | Patient-time-based NNT | Yes | Yes | No | No | NNT=1/RD | Simple proportions | No | NNT calculated as 1/RD using final rates of event and citing a median time of follow-up of 4.8 years (NNT=14 in patients with diabetes and Chronic Kidney Disease). However, a Kaplan-Meier curve is provided in the study, which should have been used (since the median follow-up is lower than the 5-years objective, at least some patients did not complete the follow-up). From the Kaplan-Meier curve, we would have 20.3% and 14.0% patients with the outcome in the atorvastatin 10 mg and 80 mg/day, respectively, at 4.8 years of follow-up and a NNT = 15.8). |
| Halonen et al. | Efficacy | Time to event | Yes | Hazard ratio | Primary outcome | NNTB | Patient-time-based NNT | Yes | Yes | No | No | NNT=1/RD | Cumulative incidence rates | Yes | Kaplan Meier curves were depicted for non-atrial fibrillation (outcome of interest). A multivariable Cox proportional hazards regression analysis model was performed to adjust for several variables. |
| Retrospective Cohort Study | | | | | | | | | | | | | | | |
| Jørgensen et al. | Safety | Binary | Yes | Odds ratio | Primary outcome | NNTH | Patient-based NNT | Yes | Yes | Yes | Yes | NNT=1/RD | Average RD | Yes | NNTH adjusted for all variables using the method and SAS macro by Bender and Vervolgyi, i.e. multiple logistic regression (average risk difference approach). |
| Smith et al. | Efficacy and Safety | Time to event | Yes | Hazard ratio | Primary outcome* | NNTB | Patient-time-based NNT | Yes | Yes | Yes** | Yes | NNT=1/RD | Cumulative incidence rates | Yes | The authors used a Cox proportional hazards regression with propensity score matching and adjustment to control for confounding. RD={[Sc(t)]^h–Sc(t)}, where Sc is the survival in control group, t is time, h is the hazard ratio. *NNT calculated only for prevention of death and CV hospitalizations. **Confidence intervals were presented only for statistically significant results. |
| Parekh et al. | Safety | Binary | Yes | Odds ratio | Primary outcome | NNTH | Patient-based NNT | No | Yes | Yes | Yes | NNT=1/RD | Average RD | Yes | Patients filling a prescription for one antimicrobial agent were considered. Method from Austin 2006 was used to compute NNT. |
| Fexer et al. | Safety | Time to event | Yes | Hazard ratio | Primary outcome | NNTH | Patient-time-based NNT | Yes | Yes | Yes | Yes | NNT=1/RD | Cumulative incidence rates | Yes | Observational study using propensity score matching. The 3.5-year observation period must be considered when interpreting NNTH results. A Kaplan–Meier estimation was used to calculate probability of exacerbation for the control and the intervention group. |
| London et al. | Efficacy | Binary | Yes | Relative risk | Primary and secondary outcomes | NNTB | Patient-based NNT | No | Yes | Yes | Yes | NNT=1/RD | Average RD | Yes | NNT were calculated as the inverse of the absolute risk reduction estimated in the propensity-score matched sample. |
| Meropol et al. | Efficacy | Binary | No | NA | Primary outcome | NNTB | Patient-based NNT | No | Yes | No | No | NNT=1/RD | Average RD | Yes | Efficacy: prevention of pneumonia. A regression model was used to calculate incidence rates and average risk differences adjusted according to several covariates. |
| Leung et al. | Safety | Time to event | Yes | Rate ratio | Primary and secondary outcomes | NNTH | Patient-time-based NNT | Yes | Yes | Yes | Yes | NNT=1/RD | Cumulative incidence rates | Yes | Incidence rates and incidence rate ratios were calculated using Poisson regression (adjusted for several covariates). A “survival” type curve (for cumulative incidences) is provided in the paper. |
| Graham et al. | Safety | Time to event | Yes | Hazard ratio | Primary outcome | NNTH | Patient-time-based NNT | Yes | Yes | Yes | Yes | NNT=1/RD | Simple proportions | No | NNT was calculated using RD between unadjusted incidence rates. Adjusted incidence rates from the Kaplan-Meier curves should have been used. For example, at one year of follow-up, NNT for the composite endpoint would be 92 from Kaplan-Meier curves, rather than 60 person-years from unadjusted incidence rates. The authors interpreted person-years as number of persons treated over one year, which is not exactly the same. |
| Wijeysundera et al. | Safety | Binary | Yes | Relative Risk | Primary outcome | NNTH | Patient-based NNT | Yes | Yes | Yes | No | NNT=1/RD | Simple proportions | Yes | NNT calculated as 1/RD (between groups for rates of death at 30 days). The outcome was analysed as a binary outcome, and not a time-to-event outcome as it often happens for survival outcomes. A non-parsimonious multivariable logistic regression model was developed. |
| Nested Case-Control Study | | | | | | | | | | | | | | | |
| Etminan et al. | Safety | Time to event | Yes | Rate ratio | Primary and secondary outcomes | NNTH | Patient-time-based NNT | No | Yes | No | Yes | Relative effect measure | Relative effect measure | Yes | Logistic regression model used to compute Rate Ratios. Patient-time based NNT can be calculated this way, but the authors should have stated that the resulting NNT equals 2500 patient-years rather than 2500 patients. |
| Bell et al. | Safety | Binary | Yes | Odds ratio | Primary outcome | NNTH | Patient-based NNT | Yes | Yes | Yes | Yes | Relative effect measure | Relative effect measure | Yes | Not clearly defined, but we assumed that the CER of 0.3% used to compute NNTH is associated with a follow-up time of 14 days. |

Legend: ¹³C-UBT: ¹³C urea breath test; ARR, absolute risk reduction; CER, control event rate; CV, cardiovascular; NA, not applicable; OR, odds ratio; NNT, number needed to treat; NNTB, number needed to treat to benefit; NNTH, number needed to treat to harm; RD, risk difference; RCT, randomized controlled trial; RR, relative risk.

**Supplemental References**

1. Jørgensen ME, Hlatky MA, KÃ¸ber L, Sanders RD, Torp-Pedersen C, Gislason GH,Jensen PF, Andersson C. Î²-Blocker-Associated Risks in Patients With Uncomplicated Hypertension Undergoing Noncardiac Surgery. JAMA Intern Med. 2015 Dec;175(12):1923-31.
2. Lenze EJ, Mulsant BH, Blumberger DM, Karp JF, Newcomer JW, Anderson SJ, Dew MA, Butters MA, Stack JA, Begley AE, Reynolds CF 3rd. Efficacy, safety, and tolerability of augmentation pharmacotherapy with aripiprazole for treatment-resistant depression in late life: a randomised, double-blind,placebo-controlled trial. Lancet. 2015 Dec 12;386(10011):2404-12.
3. Smith DH, Johnson ES, Boudreau DM, Cassidy-Bushrow AE, Fortmann SP, Greenlee RT, Gurwitz JH, Magid DJ, McNeal CJ, Reynolds K, Steinhubl SR, Thorp M, Tom JO,Vupputuri S, VanWormer JJ, Weinstein J, Yang X, Go AS, Sidney S. Comparative Effectiveness of Statin Therapy in Chronic Kidney Disease and Acute Myocardial Infarction: A Retrospective Cohort Study. Am J Med. 2015 Nov;128(11):1252.e1-1252.e11.
4. Palmerini T, Benedetto U, Bacchi-Reggiani L, Della Riva D, Biondi-Zoccai G, Feres F, Abizaid A, Hong MK, Kim BK, Jang Y, Kim HS, Park KW, Genereux P, Bhatt DL, Orlandi C, De Servi S, Petrou M, Rapezzi C, Stone GW. Mortality in patients treated with extended duration dual antiplatelet therapy after drug-eluting stent implantation: a pairwise and Bayesian network meta-analysis of randomised trials. Lancet. 2015 Jun 13;385(9985):2371-82.
5. Unger HW, Ome-Kaius M, Wangnapi RA, Umbers AJ, Hanieh S, Suen CS, Robinson LJ, Rosanas-Urgell A, Wapling J, Lufele E, Kongs C, Samol P, Sui D, Singirok D, Bardaji A, Schofield L, Menendez C, Betuela I, Siba P, Mueller I, Rogerson SJ. Sulphadoxine-pyrimethamine plus azithromycin for the prevention of low birthweight in Papua New Guinea: a randomised controlled trial. BMC Med. 2015 Jan 16;13:9.
6. Parekh TM, Raji M, Lin YL, Tan A, Kuo YF, Goodwin JS. Hypoglycemia after antimicrobial drug prescription for older patients using sulfonylureas. JAMA Intern Med. 2014 Oct;174(10):1605-12.
7. Imazio M, Brucato A, Ferrazzi P, Pullara A, Adler Y, Barosi A, Caforio AL, Cemin R, Chirillo F, Comoglio C, Cugola D, Cumetti D, Dyrda O, Ferrua S, Finkelstein Y, Flocco R, Gandino A, Hoit B, Innocente F, Maestroni S, Musumeci F, Oh J, Pergolini A, Polizzi V, Ristic A, Simon C, Spodick DH, Tarzia V, Trimboli S, Valenti A, Belli R, Gaita F; COPPS-2 Investigators. Colchicine for prevention of postpericardiotomy syndrome and postoperative atrial fibrillation: the COPPS-2 randomized clinical trial. JAMA. 2014 Sep 10;312(10):1016-23.
8. Chatterjee S, Chakraborty A, Weinberg I, Kadakia M, Wilensky RL, Sardar P, Kumbhani DJ, Mukherjee D, Jaff MR, Giri J. Thrombolysis for pulmonary embolism and risk of all-cause mortality, major bleeding, and intracranial hemorrhage: a meta-analysis. JAMA. 2014 Jun 18;311(23):2414-21.
9. Bangalore S, Makani H, Radford M, Thakur K, Toklu B, Katz SD, DiNicolantonio JJ, Devereaux PJ, Alexander KP, Wetterslev J, Messerli FH. Clinical outcomes with Î²-blockers for myocardial infarction: a meta-analysis of randomized trials. Am J Med. 2014 Oct;127(10):939-53.
10. Fexer J, Donnachie E, Schneider A, Wagenpfeil S, Keller M, Hofmann F, Mehring M. The effects of theophylline on hospital admissions and exacerbations in COPD patients: audit data from the Bavarian disease management program. Dtsch Arztebl Int. 2014 Apr 25;111(17):293-300.
11. Jonas DE, Amick HR, Feltner C, Bobashev G, Thomas K, Wines R, Kim MM, Shanahan E, Gass CE, Rowe CJ, Garbutt JC. Pharmacotherapy for adults with alcohol use disorders in outpatient settings: a systematic review and meta-analysis. JAMA. 2014 May 14;311(18):1889-900.
12. Imazio M, Belli R, Brucato A, Cemin R, Ferrua S, Beqaraj F, Demarie D, Ferro S, Forno D, Maestroni S, Cumetti D, Varbella F, Trinchero R, Spodick DH, Adler Y. Efficacy and safety of colchicine for treatment of multiple recurrences of pericarditis (CORP-2): a multicentre, double-blind, placebo-controlled, randomised trial. Lancet. 2014 Jun 28;383(9936):2232-7.
13. Lazzerini M, Martelossi S, MagazzÃ¹ G, Pellegrino S, Lucanto MC, Barabino A, Calvi A, Arrigo S, Lionetti P, Lorusso M, Mangiantini F, Fontana M, Zuin G, Palla G, Maggiore G, Bramuzzo M, Pellegrin MC, Maschio M, Villanacci V, Manenti S, Decorti G, De Iudicibus S, Paparazzo R, Montico M, Ventura A. Effect of thalidomide on clinical remission in children and adolescents with refractory Crohn disease: a randomized clinical trial. JAMA. 2013 Nov 27;310(20):2164-73.
14. Mason BJ, Quello S, Goodell V, Shadan F, Kyle M, Begovic A. Gabapentin treatment for alcohol dependence: a randomized clinical trial. JAMA Intern Med.2014 Jan;174(1):70-7.
15. London MJ, Hur K, Schwartz GG, Henderson WG. Association of perioperative Î²-blockade with mortality and cardiovascular morbidity following major noncardiac surgery. JAMA. 2013 Apr 24;309(16):1704-13.
16. Spielmans GI, Berman MI, Linardatos E, Rosenlicht NZ, Perry A, Tsai AC. Adjunctive atypical antipsychotic treatment for major depressive disorder: a meta-analysis of depression, quality of life, and safety outcomes. PLoS Med. 2013;10(3):e1001403.
17. Meropol SB, Localio AR, Metlay JP. Risks and benefits associated with antibiotic use for acute respiratory infections: a cohort study. Ann Fam Med. 2013 Mar-Apr;11(2):165-72.
18. Kayentao K, Garner P, van Eijk AM, Naidoo I, Roper C, Mulokozi A, MacArthur JR, Luntamo M, Ashorn P, Doumbo OK, ter Kuile FO. Intermittent preventive therapy for malaria during pregnancy using 2 vs 3 or more doses of sulfadoxine-pyrimethamine and risk of low birth weight in Africa: systematic review and meta-analysis. JAMA. 2013 Feb 13;309(6):594-604.
19. Liou JM, Chen CC, Chen MJ, Chen CC, Chang CY, Fang YJ, Lee JY, Hsu SJ, Luo JC, Chang WH, Hsu YC, Tseng CH, Tseng PH, Wang HP, Yang UC, Shun CT, Lin JT, Lee YC, Wu MS; Taiwan Helicobacter Consortium. Sequential versus triple therapy for the first-line treatment of Helicobacter pylori: a multicentre, open-label, randomised trial. Lancet. 2013 Jan 19;381(9862):205-13.
20. Enden T, Haig Y, KlÃ¸w NE, Slagsvold CE, Sandvik L, Ghanima W, Hafsahl G, Holme PA, Holmen LO, Njaastad AM, SandbÃ¦k G, Sandset PM; CaVenT Study Group. Long-term outcome after additional catheter-directed thrombolysis versus standard treatment for acute iliofemoral deep vein thrombosis (the CaVenT study): a randomised controlled trial. Lancet. 2012 Jan 7;379(9810):31-8.
21. Ryan NM, Birring SS, Gibson PG. Gabapentin for refractory chronic cough: a randomised, double-blind, placebo-controlled trial. Lancet. 2012 Nov 3;380(9853):1583-9.
22. Hempel S, Newberry SJ, Maher AR, Wang Z, Miles JN, Shanman R, Johnsen B, Shekelle PG. Probiotics for the prevention and treatment of antibiotic-associated diarrhea: a systematic review and meta-analysis. JAMA. 2012 May 9;307(18):1959-69.
23. Leucht S, Tardy M, Komossa K, Heres S, Kissling W, Salanti G, Davis JM. Antipsychotic drugs versus placebo for relapse prevention in schizophrenia: a systematic review and meta-analysis. Lancet. 2012 Jun 2;379(9831):2063-71.
24. Etminan M, Forooghian F, Brophy JM, Bird ST, Maberley D. Oral fluoroquinolones and the risk of retinal detachment. JAMA. 2012 Apr 4;307(13):1414-9.
25. Shah E, Kim S, Chong K, Lembo A, Pimentel M. Evaluation of harm in the pharmacotherapy of irritable bowel syndrome. Am J Med. 2012 Apr;125(4):381-93.
26. Srinivasan MG, Ndeezi G, Mboijana CK, Kiguli S, Bimenya GS, Nankabirwa V, Tumwine JK. Zinc adjunct therapy reduces case fatality in severe childhood pneumonia: a randomized double blind placebo-controlled trial. BMC Med. 2012 Feb 8;10:14.
27. Leung AA, Wright A, Pazo V, Karson A, Bates DW. Risk of thiazide-induced hyponatremia in patients with hypertension. Am J Med. 2011 Nov;124(11):1064-72.
28. Maher AR, Maglione M, Bagley S, Suttorp M, Hu JH, Ewing B, Wang Z, Timmer M, Sultzer D, Shekelle PG. Efficacy and comparative effectiveness of atypical antipsychotic medications for off-label uses in adults: a systematic review and meta-analysis. JAMA. 2011 Sep 28;306(12):1359-69.
29. Franklin ME, Sapyta J, Freeman JB, Khanna M, Compton S, Almirall D, Moore P, Choate-Summers M, Garcia A, Edson AL, Foa EB, March JS. Cognitive behavior therapy augmentation of pharmacotherapy in pediatric obsessive-compulsive disorder: the Pediatric OCD Treatment Study II (POTS II) randomized controlled trial. JAMA. 2011 Sep 21;306(11):1224-32.
30. Imazio M, Brucato A, Cemin R, Ferrua S, Belli R, Maestroni S, Trinchero R, Spodick DH, Adler Y; CORP (COlchicine for Recurrent Pericarditis) Investigators. Colchicine for recurrent pericarditis (CORP): a randomized trial. Ann Intern Med. 2011 Oct 4;155(7):409-14.
31. Preiss D, Seshasai SR, Welsh P, Murphy SA, Ho JE, Waters DD, DeMicco DA, Barter P, Cannon CP, Sabatine MS, Braunwald E, Kastelein JJ, de Lemos JA, Blazing MA, Pedersen TR, Tikkanen MJ, Sattar N, Ray KK. Risk of incident diabetes with intensive-dose compared with moderate-dose statin therapy: a meta-analysis. JAMA. 2011 Jun 22;305(24):2556-64.
32. Brinks A, van Rijn RM, Willemsen SP, Bohnen AM, Verhaar JA, Koes BW, Bierma-Zeinstra SM. Corticosteroid injections for greater trochanteric pain syndrome: a randomized controlled trial in primary care. Ann Fam Med. 2011 May-Jun;9(3):226-34.
33. Shamliyan TA, Johnson JR, MacDonald R, Shaukat A, Yuan JM, Kane RL, Wilt TJ. Systematic review of the literature on comparative effectiveness of antiviral treatments for chronic hepatitis B infection. J Gen Intern Med. 2011 Mar;26(3):326-39.
34. Coker TR, Chan LS, Newberry SJ, Limbos MA, Suttorp MJ, Shekelle PG, Takata GS. Diagnosis, microbial epidemiology, and antibiotic treatment of acute otitis media in children: a systematic review. JAMA. 2010 Nov 17;304(19):2161-9.
35. Zinman B, Harris SB, Neuman J, Gerstein HC, Retnakaran RR, Raboud J, Qi Y, Hanley AJ. Low-dose combination therapy with rosiglitazone and metformin to prevent type 2 diabetes mellitus (CANOE trial): a double-blind randomised controlled study. Lancet. 2010 Jul 10;376(9735):103-11.
36. Graham DJ, Ouellet-Hellstrom R, MaCurdy TE, Ali F, Sholley C, Worrall C, Kelman JA. Risk of acute myocardial infarction, stroke, heart failure, and death in elderly Medicare patients treated with rosiglitazone or pioglitazone. JAMA. 2010 Jul 28;304(4):411-8.
37. Bell CM, Hatch WV, Fischer HD, Cernat G, Paterson JM, Gruneir A, Gill SS, Bronskill SE, Anderson GM, Rochon PA. Association between tamsulosin and serious ophthalmic adverse events in older men following cataract surgery. JAMA. 2009 May 20;301(19):1991-6.
38. Testa L, van Gaal WJ, Biondi-Zoccai GG, Abbate A, Agostoni P, Bhindi R, Banning AP. Repeat thrombolysis or conservative therapy vs. rescue percutaneous coronary intervention for failed thrombolysis: systematic review and meta-analysis. QJM. 2008 May;101(5):387-95.
39. Kenyon S, Pike K, Jones DR, Brocklehurst P, Marlow N, Salt A, Taylor DJ. Childhood outcomes after prescription of antibiotics to pregnant women with spontaneous preterm labour: 7-year follow-up of the ORACLE II trial. Lancet. 2008 Oct 11;372(9646):1319-27.
40. Wijeysundera DN, Beattie WS, Austin PC, Hux JE, Laupacis A. Epidural anaesthesia and survival after intermediate-to-high risk non-cardiac surgery: a population-based cohort study. Lancet. 2008 Aug 16;372(9638):562-9.
41. Shepherd J, Kastelein JP, Bittner VA, Carmena R, Deedwania PC, Breazna A, Dobson S, Wilson DJ, Zuckerman AL, Wenger NK; Treating to New Targets Steering Committee and Investigators. Intensive lipid lowering with atorvastatin in patients with coronary artery disease, diabetes, and chronic kidney disease. Mayo Clin Proc. 2008 Aug;83(8):870-9.
42. Christensen R, Kristensen PK, Bartels EM, Bliddal H, Astrup A. Efficacy and safety of the weight-loss drug rimonabant: a meta-analysis of randomised trials. Lancet. 2007 Nov 17;370(9600):1706-13.
43. Green H, Paul M, Vidal L, Leibovici L. Prophylaxis of Pneumocystis pneumonia in immunocompromised non-HIV-infected patients: systematic review and meta-analysis of randomized controlled trials. Mayo Clin Proc. 2007 Sep;82(9):1052-9.
44. Bridge JA, Iyengar S, Salary CB, Barbe RP, Birmaher B, Pincus HA, Ren L, Brent DA. Clinical response and risk for reported suicidal ideation and suicide attempts in pediatric antidepressant treatment: a meta-analysis of randomized controlled trials. JAMA. 2007 Apr 18;297(15):1683-96.
45. Halonen J, Halonen P, JÃ¤rvinen O, Taskinen P, Auvinen T, Tarkka M, HippelÃ¤inen M, Juvonen T, Hartikainen J, Hakala T. Corticosteroids for the prevention of atrial fibrillation after cardiac surgery: a randomized controlled trial. JAMA. 2007 Apr 11;297(14):1562-7.
46. Leontiadis GI, Sharma VK, Howden CW. Proton pump inhibitor therapy for peptic ulcer bleeding: Cochrane collaboration meta-analysis of randomized controlled trials. Mayo Clin Proc. 2007 Mar;82(3):286-96.
47. Dentali F, Douketis JD, Gianni M, Lim W, Crowther MA. Meta-analysis: anticoagulant prophylaxis to prevent symptomatic venous thromboembolism in hospitalized medical patients. Ann Intern Med. 2007 Feb 20;146(4):278-88.
48. Rovers MM, Glasziou P, Appelman CL, Burke P, McCormick DP, Damoiseaux RA, Gaboury I, Little P, Hoes AW. Antibiotics for acute otitis media: a meta-analysis with individual patient data. Lancet. 2006 Oct 21;368(9545):1429-35.
49. Hollingsworth JM, Rogers MA, Kaufman SR, Bradford TJ, Saint S, Wei JT, Hollenbeck BK. Medical therapy to facilitate urinary stone passage: a meta-analysis. Lancet. 2006 Sep 30;368(9542):1171-9.
50. Bongartz T, Sutton AJ, Sweeting MJ, Buchan I, Matteson EL, Montori V. Anti-TNF antibody therapy in rheumatoid arthritis and the risk of serious infections and malignancies: systematic review and meta-analysis of rare harmful effects in randomized controlled trials. JAMA. 2006 May 17;295(19):2275-85.
51. Spiegel BM, Farid M, Dulai GS, Gralnek IM, Kanwal F. Comparing rates of dyspepsia with Coxibs vs NSAID+PPI: a meta-analysis. Am J Med. 2006 May;119(5):448.e27-36.
